# Supplementary material for: Template Learning: Deep learning with domain randomization for particle picking in cryo-electron tomography
Source: Nat Commun. 2025 Oct 3;16:8833. doi: 10.1038/s41467-025-63895-0 (PMC12494793; doi:10.1038/s41467-025-63895-0)
Supplement: Supplementary file 1 — Supplementary Information [file 41467_2025_63895_MOESM1_ESM.pdf]

Template Learning: Deep Learning with Domain Randomization for Particle Picking in Cryo-Electron Tomography

Mohamad Harastani[1,2,*], Gurudatt Patra[1], Charles Kervrann[3], Mikhail Eltsov[1,*]

[1]Department of Integrated Structural Biology, Institute of Genetics and Molecular and Cellular Biology, Illkirch, France

[2]Institut Pasteur, Université Paris Cité, Image Analysis Hub (IAH), Paris, France

[3]Inria Center at University of Rennes, SAIRPICO Team, Cellular and Chemical Biology Unit, U1143 INSERM, UMR3666 CNRS, Institut Curie, PSL Research University, Campus universitaire de Beaulieu, Rennes Cedex, France

*Correspondence: Mohamad Harastani and Mikhail Eltsov {mohamad.harastani@pasteur.fr, mikhail.eltsov@igbmc.fr}

Supplementary Information File

Table of Contents

Template Learning Variations 2
Adapting Template Learning to different domains 6
Supplementary Figures and Tables 8
Supplementary Note 1: Masking Cryo-ET Annotations Based on a Region of Interest 18
Supplementary Note 2: Descriptive Statistics and Statistical Tests of Template Learning Variations 20
Supplementary Note 3: Impact of Target-Distractor Similarity on Template Learning Performance 29
Supplementary Note 4: Subtomogram Averaging of Ribosomes In Situ: Template Learning vs. DeePiCt and Expert Annotations 31
Supplementary Note 5: Template Learning Shows Advantages Over Supervised Deep Learning and Template Matching for Annotating Nucleosomes In Situ 38

Template Learning Variations

We introduced several concepts in the Template Learning data simulation workflow, demonstrating that, when combined, they achieve state-of-the-art performance for supervised deep learning in annotating ribosomes within *in situ* cryo-ET tomograms. To study the significance of each proposed concept and explore the potential for integrating various modifications, we conducted additional experiments, which we have organized into the following four categories:

Incorporating multiple atomic template structures and flexible variations

In three experiments, we progressively reduced the structural variability of input templates while keeping the remaining parameters of the method unchanged. Our motivation behind this series of experiments is to guide potential users regarding the outcomes of employing multiple template structures and integrating flexible variations. These experiments can be particularly useful to future studies, recognizing that, for certain biomolecules, the presence of multiple structures and the feasibility of simulating molecular mechanics may vary.

In one experiment, we retained from the templates a single structure and its flexible variations. In a second experiment, we retained the six structures while excluding the flexible variations. Finally, in a third experiment, we retained only a single structure, eliminating other structures and all flexible variations. The trained deep learning models resulting from the Template Learning workflows of these three experiments were used to analyze the dataset as presented previously.

Fig.4(B-D, I) in the main text depicts the curves for Recall and Precision, and boxplots for F_1 scores for the three experiments (see Supplementary Note 2 for more detail). Notably, employing a single structure with flexible variations yielded comparable results to employing multiple structures without flexible variations. We can infer from these findings that introducing artificial flexible variations can serve as an effective compensatory mechanism for the absence of multiple PDBs. This becomes particularly relevant in studies where only a limited number of atomic structures, or even just a single structure, is publicly accessible. Both approaches (i.e., a single PDB with flexibility and multiple PDBs without flexibility) showed a slight adverse impact on the median F_1 score of the aforementioned typical template learning workflow, of approximately 5%. However, they still demonstrated comparable performance to previously reported results from supervised deep learning on annotated experimental data (compare Fig.4I/B-C and Fig.4J).

In contrast, employing a single template without any flexible variations yielded a stricter model characterized by higher Precision and lower Recall—translating to fewer false

positives at the cost of more false negatives (see Supplementary Fig. 3 for the output segmentation maps). Overall, the use of a single PDB template led to a relative reduction in the median F_1 score by approximately 10%. Nevertheless, it maintains a significant advantage over traditional template matching (compare Fig.4I/D and Fig.4J/Template matching).

Incorporating distractors

In contrast to our proposed Template Learning method, previous studies did not emphasize the incorporation of a background combining a comprehensive array of dissimilar distractors. In [1,2], no distractors were employed in the data simulation to train deep learning models on subtomogram segmentation. Two recent studies [3,4] utilized a small set of objects, serving as analogs to distractors in the simulation of data for training deep neural networks in regression tasks, including signal restoration and segmentation. These objects included gold fiducials, actin bundles, vesicles, and randomly placed small spheres that were relatively sparsely distributed in the volume. Our strategy is principally different; it utilizes other real biomolecules as distractors for a simple approximation of the realistic crowding of biomolecular environments.

To investigate the contribution of employing as many as 100 dissimilar distractors to annotate only a single target structure on the state-of-the-art performance of Template Learning, we conducted two additional experiments on distractors while keeping other method parameters unchanged. In one experiment, we removed all distractors from the simulated tomograms, following a methodology similar to that outlined in [1]. Fig.4(E, I) depicts the curves for Recall and Precision, and boxplots for F_1 scores for this experiment, revealing that the absence of distractors during Template Learning results in models that are not specific to picking targets and can mistake other objects for targets in experimental tomograms. Hence, removing distractors from the simulated data results in hallucinations in the output segmentations leading to a high rate of false positives (refer to Supplementary Fig. 3). We also addressed the effects of accidental partial target-distractor similarity. In this experiment, all distractors were retained, but approximately 10% of target molecules were mislabeled as distractors during training. We observed no significant decline in model performance (see Supplementary Note 3 for details).

In a subsequent experiment, we explored if there are benefits of using many distractors compared to a limited number, similar to previous approaches [3,4]. Subsequently, we reintroduced three distractors—small (PDB 1S3X), medium (PDB 5A20), and large (PDB 6UP6) proteins—while maintaining the previously established volumetric density ratio balance between distractors and templates in the simulated tomograms. Fig.4(F, I) depicts the curves for Recall and Precision, and boxplots for F_1 scores for this experiment.

These results show that limiting the variability of distractors had a serious adverse impact on the picking Precision.

These experiments suggest that exposing the deep learning model to a diverse range of unwanted structures (i.e., distractors) alongside the target structures (i.e., templates) is essential for the best performance.

Simulating crowding

To investigate the contribution of simulating high crowding, (i.e., using the Tetris algorithm introduced in this work) to the state-of-the-art performance of Template Learning, we aimed to design an experiment involving crowding reduction. Consequently, we continued our investigation of the crowding reduction impact on Template Learning by adapting the Tetris algorithm to systematically generate crowding levels aiming for 25-35% crowding relative to the original settings while keeping all other parameters unchanged (to see how crowding can be controlled in the Tetris algorithm, see the corresponding Methods section).

Fig.4(G, I) depicts the curves for Recall and Precision, and boxplots for F_1 scores for this experiment, revealing that the deep learning model trained on a simulated dataset with less crowding resulted in a 20-25% decrease in the median F_1 score.

Using a volumetric template

Template Learning utilizes fully atomic structures and physics-based simulations to train deep learning models as alternatives to volume templates in traditional cryo-ET template matching. An advantage of employing fully atomic structures is the utilization of rapid molecular mechanics simulations using NMA. Particularly, our strategy involves coarse-graining the structures before NMA, and interpolating the resulting motions to the original fully-atomic model (refer to the NMA section in Methods for further details).

However, using volumetric templates, in the context of particle picking, can be advantageous in specific scenarios, particularly when an initial structure can be derived from the dataset. For instance, a subtomogram average at low resolution derived from a partially annotated dataset can serve as an initial template to annotate more particles in the dataset, enabling the generation of a higher-resolution average.

Consequently, we explored a method to incorporate a volume as a template in the physics-based simulation, rather than relying solely on atomic structures. Existing literature demonstrates the feasibility of generating pseudoatomic models from volumes, primarily by estimating the volume using a set of 3D Gaussian functions [5,6]. The mean and standard deviations of these 3D Gaussians are calculated in a way that the

pseudoatomic model, when converted back to a volume (i.e., via summation of the contribution of each 3D Gaussian to each voxel), estimates the volume with low error (e.g., 5%). However, advanced cryo-ET physics-based simulators (e.g., Parakeet, used in this work), do make use of the characteristics of these 3D Gaussians in the simulations, instead, they use electron-atom interactions to model the image formation in the TEM. Meaning, pseudoatomic structures based on previous works do not generate the expected contrast when used in such physics-based simulators.

Hence, we devised a two-stage algorithm for real-time conversion of volumes into pseudoatomic models that allows tuning the contrast by substituting some pseudoatoms with actual phosphorus atoms (see Supplementary Fig. 4 for illustration and Methods for details).

To proceed with investigating the effect of using a volume as a template for ribosome annotations, we employed the atomic structure of a ribosome (PDB 4UG0) to generate a volume at 2 nm resolution using the Eman2 [7] pdb2mrc software. It is noteworthy that in the study [8] where the dataset (EMPIAR-10988) was published, a sub-nanometer subtomogram average was obtained. Here, we explored a hypothetical scenario where a 2 nm resolution initial average is obtained by averaging a partially annotated subset of the original dataset and investigated if the Template Learning workflow can employ this structure for annotating more ribosomes. Subsequently, we transformed this volume into a pseudoatomic structure, following the steps outlined in the corresponding Methods section. As part of this conversion process, we introduced a substitution, replacing one-third of the pseudoatoms with phosphorus atoms to approximate the contrast of ribosomes observed in experimental data (Supplementary Fig. 4).

We employed this pseudoatomic model to generate simulations using the Template Learning workflow, by replacing the atomic structures that were previously used as input, keeping the remaining parameters unchanged.

Fig.4(H, I) depicts the curves for Recall and Precision, and boxplots for F_1 scores for this experiment, indicating that the deep learning model trained on a simulated dataset with the mentioned pseudoatomic structure exhibits a lower Precision and a higher Recall compared to training with an actual atomic structure. We interpret these findings suggesting that lower resolution template structure (2 nm instead of atomic resolution) removed certain details from the simulation, resulting in a deep learning model that is more permissive but less precise in its predictions. Nonetheless, the F_1 score of this experiment shows a 27% increase relative to traditional template matching, despite that both methods used a similar prior (i.e., a single volume ribosome template).

Adapting Template Learning to different domains

In the preceding section, we benchmarked Template Learning for annotating ribosomes in a close-to-focus VPP cryo-ET dataset *in situ*. Hence, the previous Template Learning workflow for ribosome annotation involved simulating data using VPP close-to-focus, consistent with the data analyzed.

In addition to VPP data, the dataset (EMPIAR-10988) contains an additional set of 10 tomograms acquired with defocus (DEF tomograms) and without VPP. In the original study [8], the DeePiCt deep learning model was trained on the experimental VPP tomograms and subsequently evaluated on DEF tomograms pre-processed with spectrum matching (SM). SM enhanced the contrast of the DEF dataset to resemble that of the VPP dataset, achieved by extracting a target spectrum from a VPP tomogram and applying it to the DEF tomograms (see Supplementary Fig. 5). This approach was referred to as the DeePiCt "cross-domain" experiment.

We conducted two experiments to explore the impact of the data domain on Template Learning. In the first experiment, we set up a Template Learning workflow targeting the DEF domain. We accomplished this by generating a simulated dataset using a procedure identical to the one outlined in the preceding section except for excluding VPP simulations and employing a different range of defocus values (refer to Supplementary Table 1 for details). This simulated dataset was used to train and benchmark DeepFinder on annotating ribosomes within the DEF tomograms, without undergoing SM preprocessing. In the second experiment, we utilized the DeepFinder model trained on Template Learning VPP simulations to annotate ribosomes within the DEF dataset that had undergone SM preprocessing.

Fig.5 depicts the curves for Recall and Precision, and boxplots for F_1 scores for the two aforementioned experiments, compared to the previously reported result of the DeePiCt cross-domain experiment. Both of our experiments yield comparable outcomes, outperforming the DeePiCt cross-domain experiment by more than 10% on the median F_1 score. Notably, the Template Learning-trained model for DEF data exhibited a higher Precision curve and ultimately achieved a higher F_1 score, particularly when no mask (cytosol mask) was applied.

The first experiment shows that the Template Learning workflow can be directly adapted to the DEF domain avoiding the need for data preprocessing such as SM. The second experiment shows that if a model trained on the Template Learning simulations of the VPP domain exists, it can be reused in the cross-domain (i.e., the DEF domain) after SM, and achieves a similar performance without repeating the entire Template Learning workflow. Additional validation of Template Learning performance using subtomogram

averaging (STA) is detailed in Supplementary Note 4. We show that the STA of peaks identified by Template Learning resulted in a slight improvement in the resolution of the average compared to the ones extracted by DeePiCt. Additionally, Template Learning identified true positives missed by the expert annotations (see Supplementary Note 4 for details).

Supplementary Figures and Tables

Supplementary Fig. 1: The list of distractors.

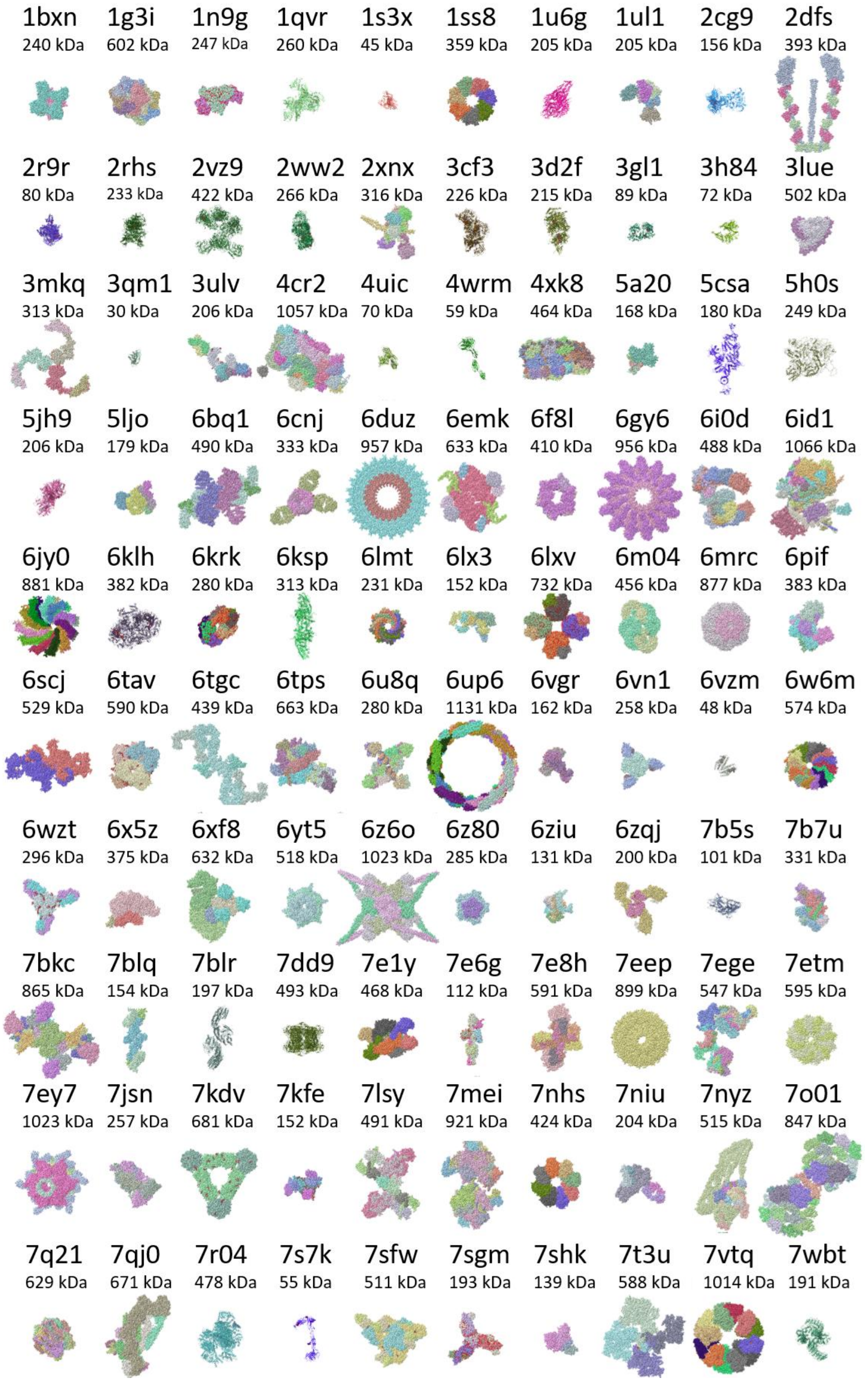

Template Learning employs a list of 100 dissimilar protein assemblies, termed distractors, used for domain-randomized cryo-ET data simulations. In this figure, the

distractors are displayed with corresponding PDB IDs and molecular weights. Display of these structures was done using ChimeraX [9].

Supplementary Fig. 2: Example of synthetic data based on the Template Learning workflow for supervised deep learning on ribosome annotation.

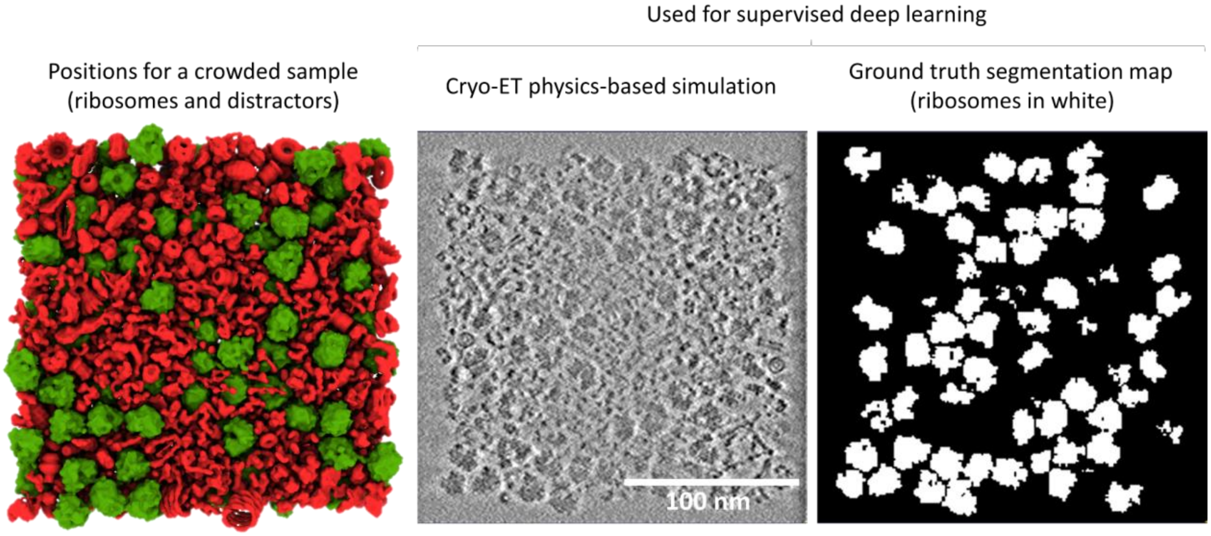

Left: the positions for a crowded sample of size 3072x3072x1024 Å^3 determined using the Tetris algorithm for ribosomes (in green) and distractors (in red), which are fed to Parakeet (cryo-ET physics-based simulator). Right: a central slice of simulated data and corresponding ribosome segmentations using VPP close-to-focus parameters.

Supplementary Fig. 3: Segmentation maps resulting from different Template Learning variations for ribosome annotations.

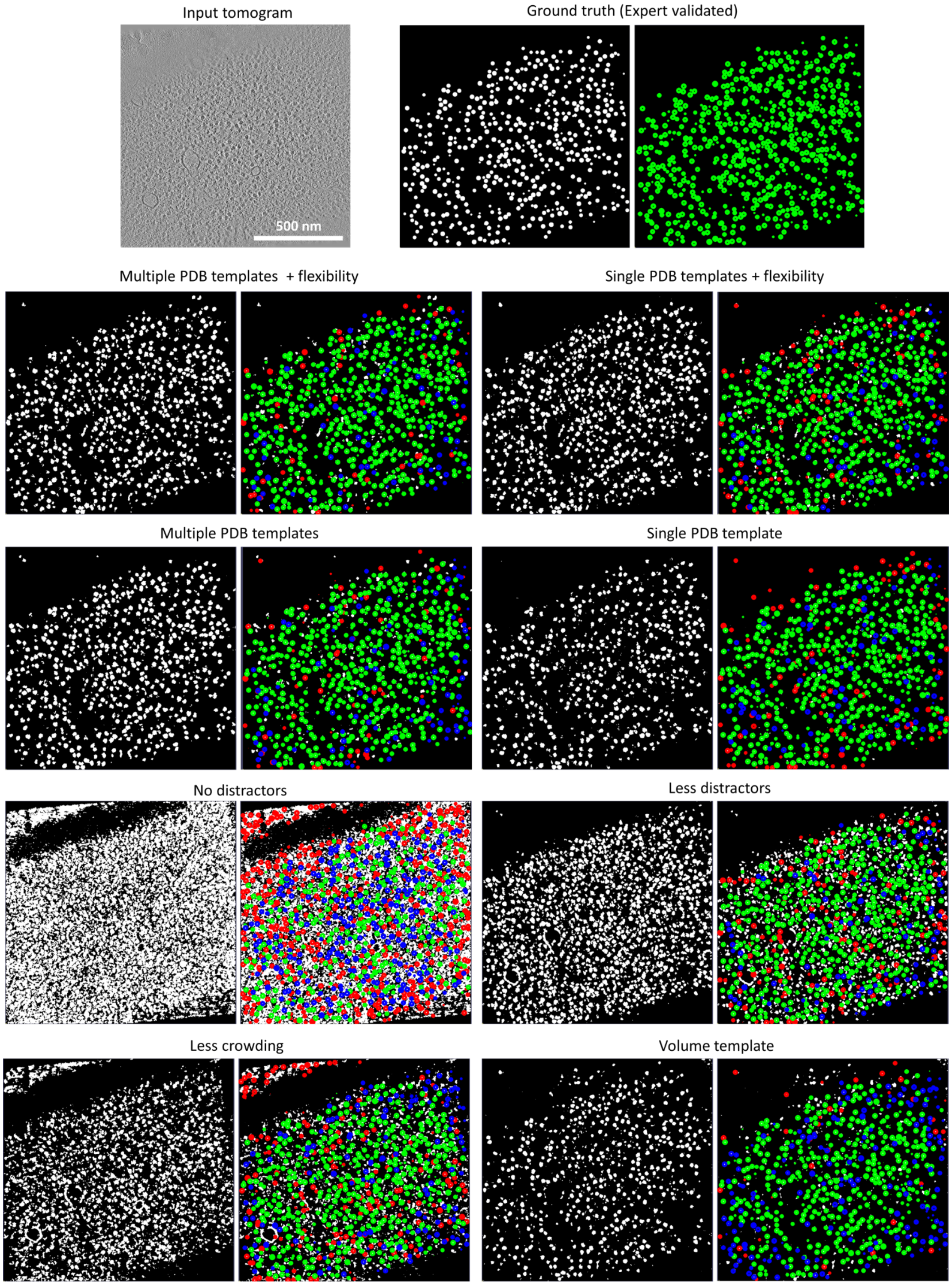

Central slice of a VPP tomogram from EMPIAR-10988 with its expert-validated (ground truth) ribosome segmentation and the output segmentation maps using the different

Template Learning variations presented in **Fig.4**. The segmentation maps are displayed in their raw form (left) and color-coded (right) after extracting annotations from the centroids of the segments using meanshift with a clustering radius of 10 voxels and applying a segment threshold based on the model's optimal performance for each experiment (balancing precision and recall). In the color-coded segmentation maps, green represents true positives, red represents false positives, blue represents false negatives, and white regions represent signals that did not influence the annotation, typically because they were smaller than the optimal threshold.

Supplementary Fig. 4: Template Learning can use volumes as templates via volume-to-pseudoatoms conversion.

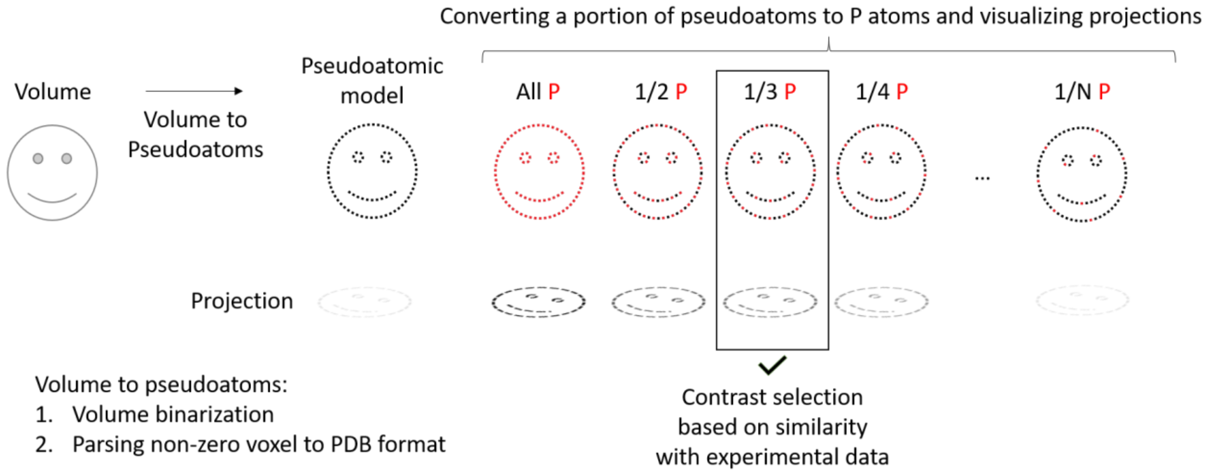

Volume to pseudoatomic model conversion algorithm to produce structures that can be contrast-tuned for usage in physics-based cryo-ET simulators (Parakeet). The method starts by binarizing the volume, a process akin to creating a tight mask through low-pass filtering and thresholding. All non-zero voxels of the binarized volume are parsed into "pseudoatoms", represented in PDB file format utilizing "DENS" entries as atom types. The generated pseudoatomic structure can be used directly for cryo-ET simulations in Parakeet, but it does not necessarily produce a similar contrast to experimental data as when using fully atomic structures. Therefore, the method allows replacing gradually a portion of the pseudoatoms with phosphorus atoms to emulate more contrasted simulated projections. The optimal proportion is determined by the similarity in contrast, comparing the resulting projections to experimental data. The resultant pseudoatomic model is used for further Template Learning simulations.

Supplementary Fig. 5: Spectrum matching applied to enhance the contrast of an input tomogram obtained with defocused cryo-ET without VPP.

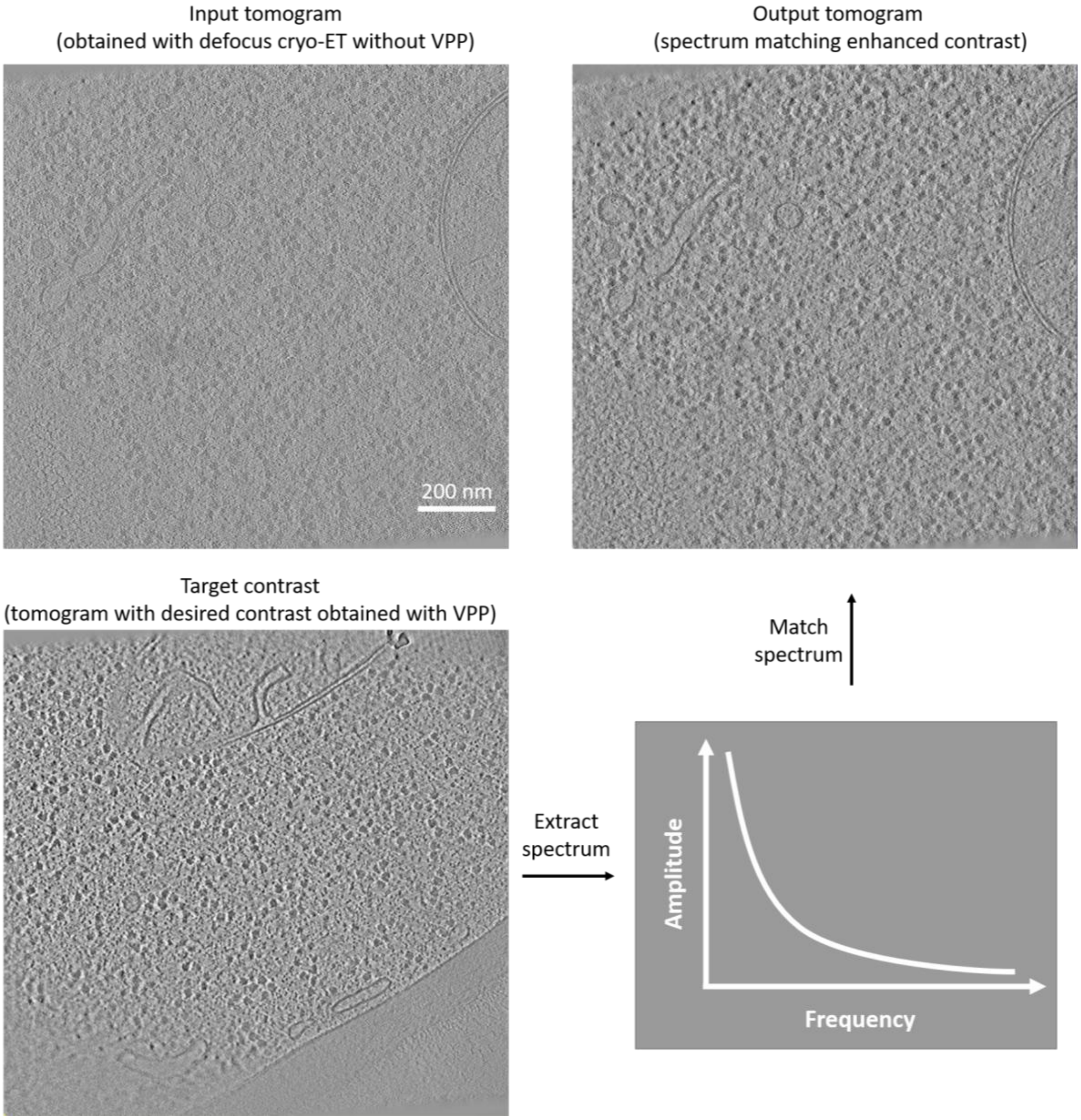

A target spectrum is extracted from a tomogram obtained with VPP, and is applied to enhance the contrast of another tomogram obtained with defocus cryo-ET without VPP, via spectrum matching as proposed in [8]. Input and target tomograms are sources from EMPIAR-10988.

Supplementary Fig. 6: Two experiments of post-alignment classification for nucleosome particles annotated with Template Learning workflow.

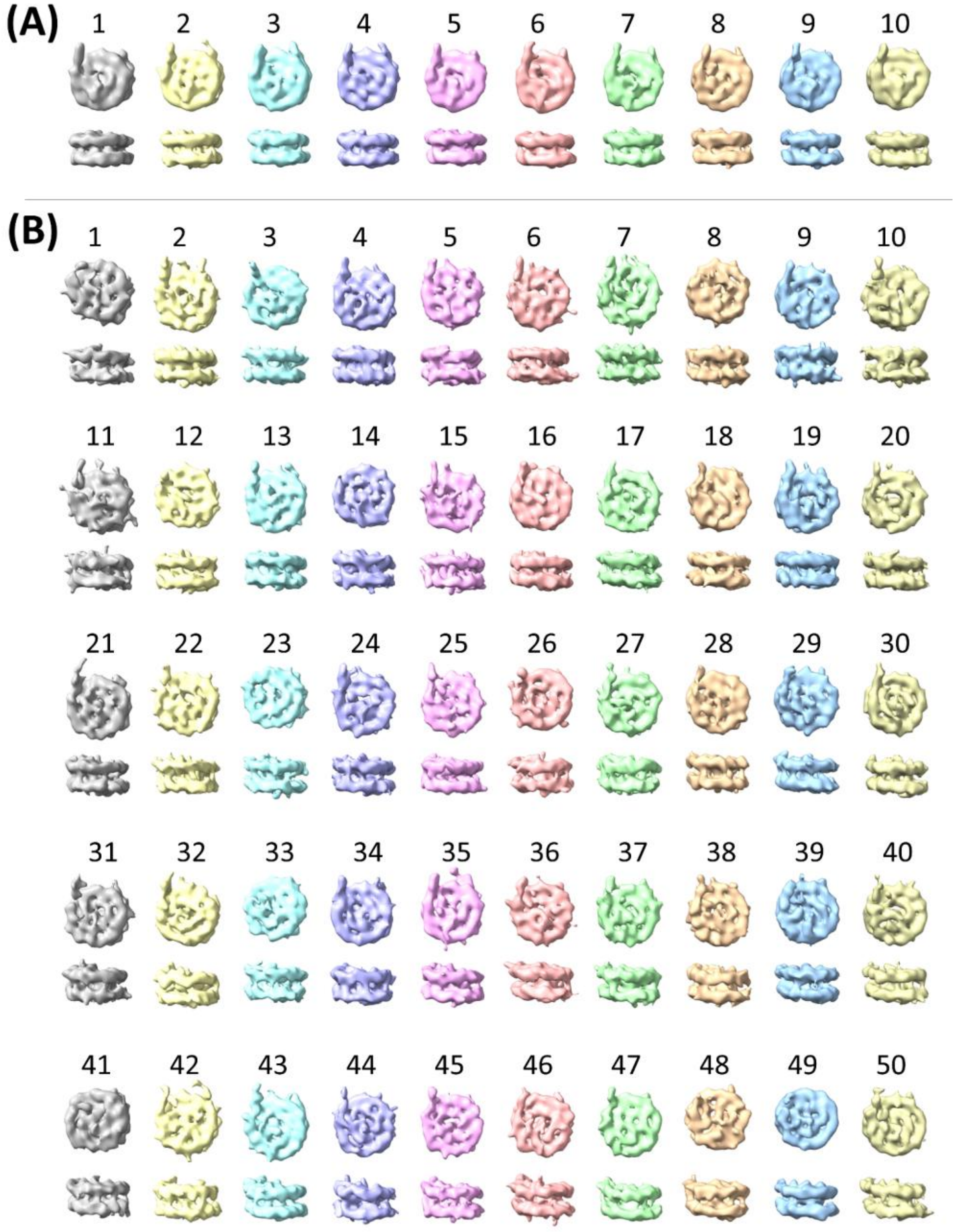

A Classification into 10 classes. **B** Classification into 50 classes. Both classification experiments were done in Relion V4 [10].

Supplementary Fig. 7: Analysis of the nucleosome subtomogram average obtained via Template Learning annotations.

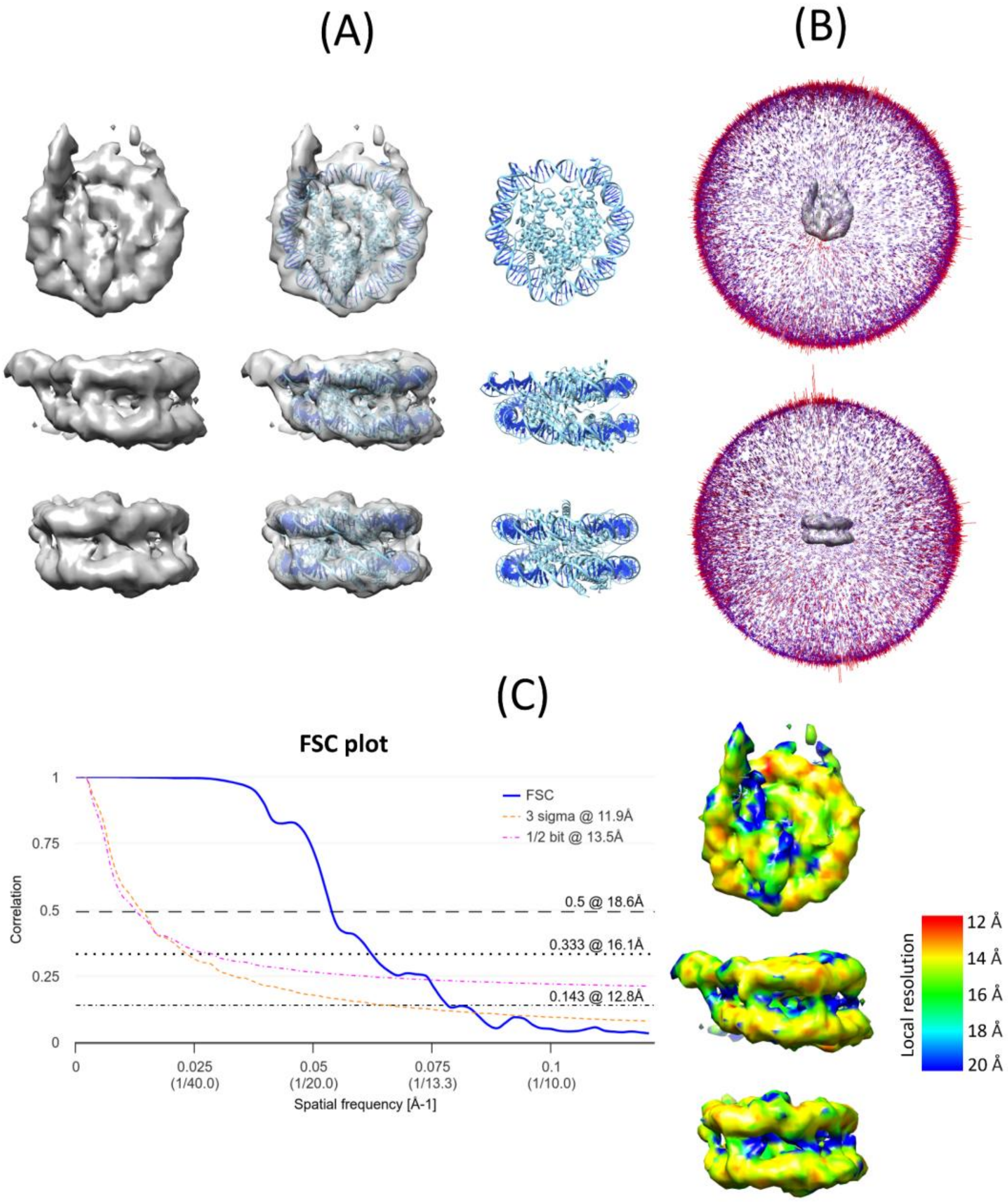

A The nucleosome average compared to an available structure (PDB ID: 2CV5). Left: the subtomogram average. Middle: atomic structure docked in the subtomogram average displayed at 50% transparency. Right: atomic structure. **B** Angular distribution of the particles contributing to the global average. **C** Analysis of the resolution of the average. Left: Fourier Shell Correlation curves. Right: local resolution analysis using ResMap [11].

Supplementary Fig. 8: Template matching for nucleosome picking can only extract certain views at an adequate Precision.

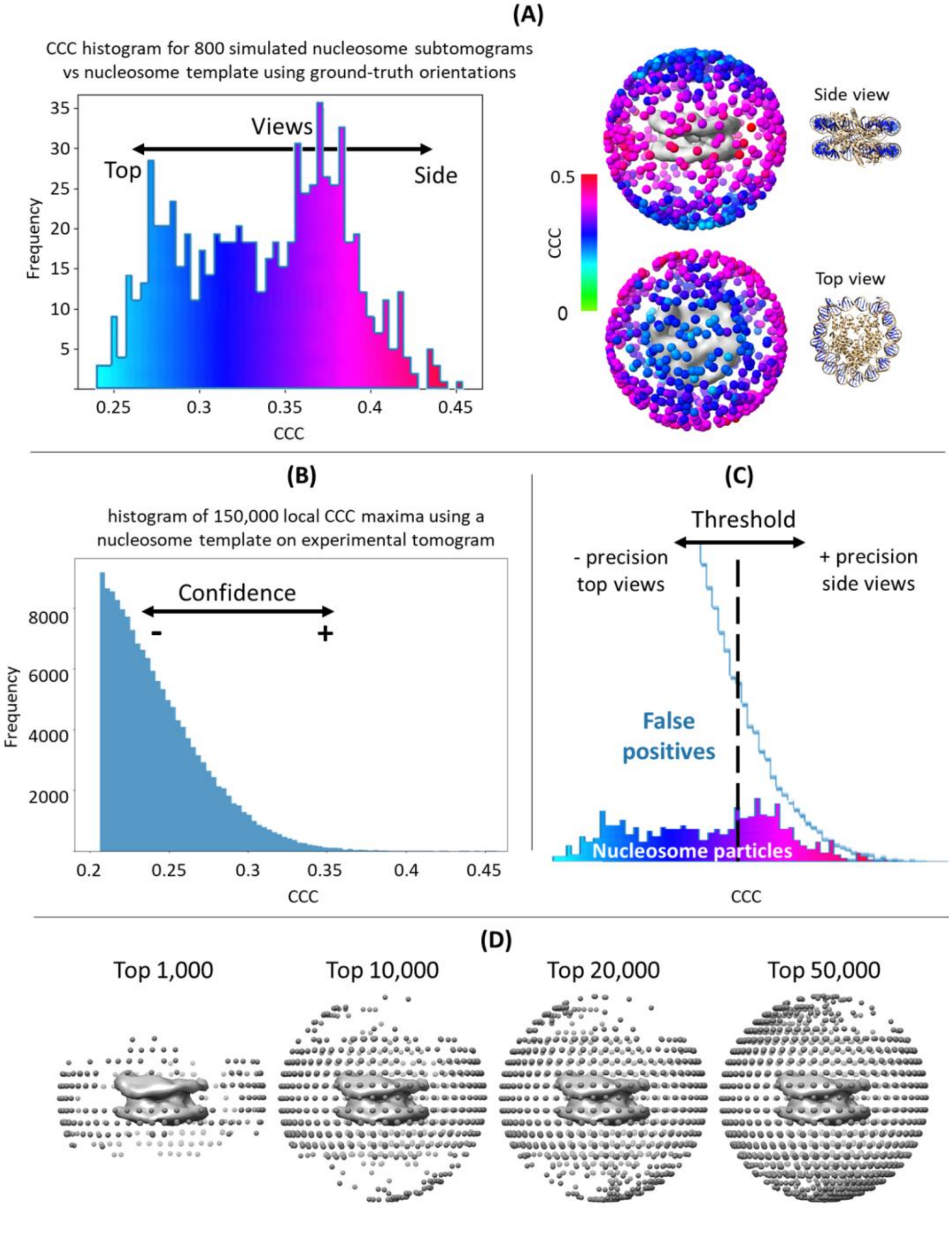

In this work, we utilized template matching and Template Learning techniques to annotate nucleosomes in cryo-electron tomograms. The angular distribution obtained through averaging of template matching-based annotated particles was found anisotropic. Leveraging simulations, we explain this phenomenon. **A** Constrained

cross-correlation (CCC) of nucleosome template and simulated nucleosome subtomograms as a function of the angular distribution. Left: CCC histogram of a nucleosome template and ~800 simulated nucleosome subtomograms. The simulated subtomograms were obtained using ±60 degree tilt range with a 2-degree step, 2 microns defocus, and 120 e-/Å dose symmetric scheme using Parakeet. The template was used with the ground truth angular assignment before calculating the constrained cross-correlation (CCC). Right: angular distribution colored based on the CCC values showing that side views have higher CCC compared to top views. The nucleosome atomic structure (PDB 2PYO) was oriented similarly to the volume template shown in the middle. **B** CCC histogram as a result of template matching using PyTom on an experimental tomogram. **C** Illustration based on combining the results of **A** and **B** showing that thresholding based on the CCC value can extract only side views at an adequate precision while lowering the threshold increases the number of extracted top views but at the expense of extracting many false positives. **D** Angular distribution of template matching picked particles at different thresholds in **A** showing that the particles with the highest CCC scores correspond to nucleosome side views.

Supplementary Table 1: Cryo-ET simulation parameters in Template Learning.

The parameters used for domain randomization simulations in this article were based on a combination of the parameters listed in this table.

Parameter	Values
Defocus for close-to-focus VPP [microns]	0, -0.5, -1
Defocus without VPP [microns]	-2.5, -3.25, -4
Electron dose [$e^-/Å^2$]	75, 150
Tilting range [deg]	[-40, 40], [-60, 60]
Tilting step [deg]	2, 4
Ice density [g/cm^3]	0.9, 1.1

Supplementary Table 2: Parameters used for training DeepFinder on Template Learning simulations.

Default hyperparameters used to train DeepFinder for the different particle picking experiments.

Parameter	Values
Number of epochs	100
Number of steps	100
Shifting for data augmentation [vox]	13
Patch size [vox^3]	48
Batch size	25

Supplementary Note 1: Masking Cryo-ET Annotations Based on a Region of Interest

To benchmark variations in the Template Learning approach and compare it with other methods, we used the publicly available dataset for S. pombe ribosomes *in situ* (EMPIAR-10988, VPP). In addition to tomograms, this dataset includes cytosol masks, which help exclude regions that cannot contain ribosomes, such as nucleus and cytoplasmic vesicles, thereby reducing false positives. We validated our method with and without masking to quantify how many false positives are annotated outside the region of interest. This supplementary note provides an example of the effectiveness of cytosol masking in removing false positives, along with guidance for generating these masks for future applications on new datasets.

Supplementary Fig. 9 illustrates the impact of cytosol masking for facilitating the annotation of cytosolic ribosomes. Masking removes the false positives outside the region of interest (i.e., the cytoplasm). False positives outside the region of interest are typically noise, other molecules, membranes, or ice contamination. In some cases, false positives may correspond to molecules that share structural similarity (e.g., subunits in common) with the target particles, such as the pre-ribosomal particles in the nucleolus region or captured in nucleoplasm during their transport to cytoplasm, that are removed by cytosol masking.

Cytosol masks can be manually generated using tools like SuRVoS [12] (Super-Region Volume Segmentation) workbench, which offers preprocessing steps through filtering and super voxel generation to facilitate manual segmentation. Alternatively, trained deep neural network models such as DeePiCt [8], a 2D convolutional neural network (CNN), can replicate these masks with a median F_1 score of 95–97%, depending on the domain. Such segmentation accuracy is often sufficient for large datasets used in subtomogram averaging or can serve as a starting point, followed by manual refinement in smaller datasets.

Beyond mask generation, certain software packages, such as the EMAN2 [7] tomography particle viewer, offer simpler approaches for reducing false positives outside the region of interest. The EMAN2 viewer provides a “brush eraser” tool that allows users to bulk-remove unwanted particles outside the region of interest, allowing the process of manually eliminating false positives to be more efficient than removing them individually.

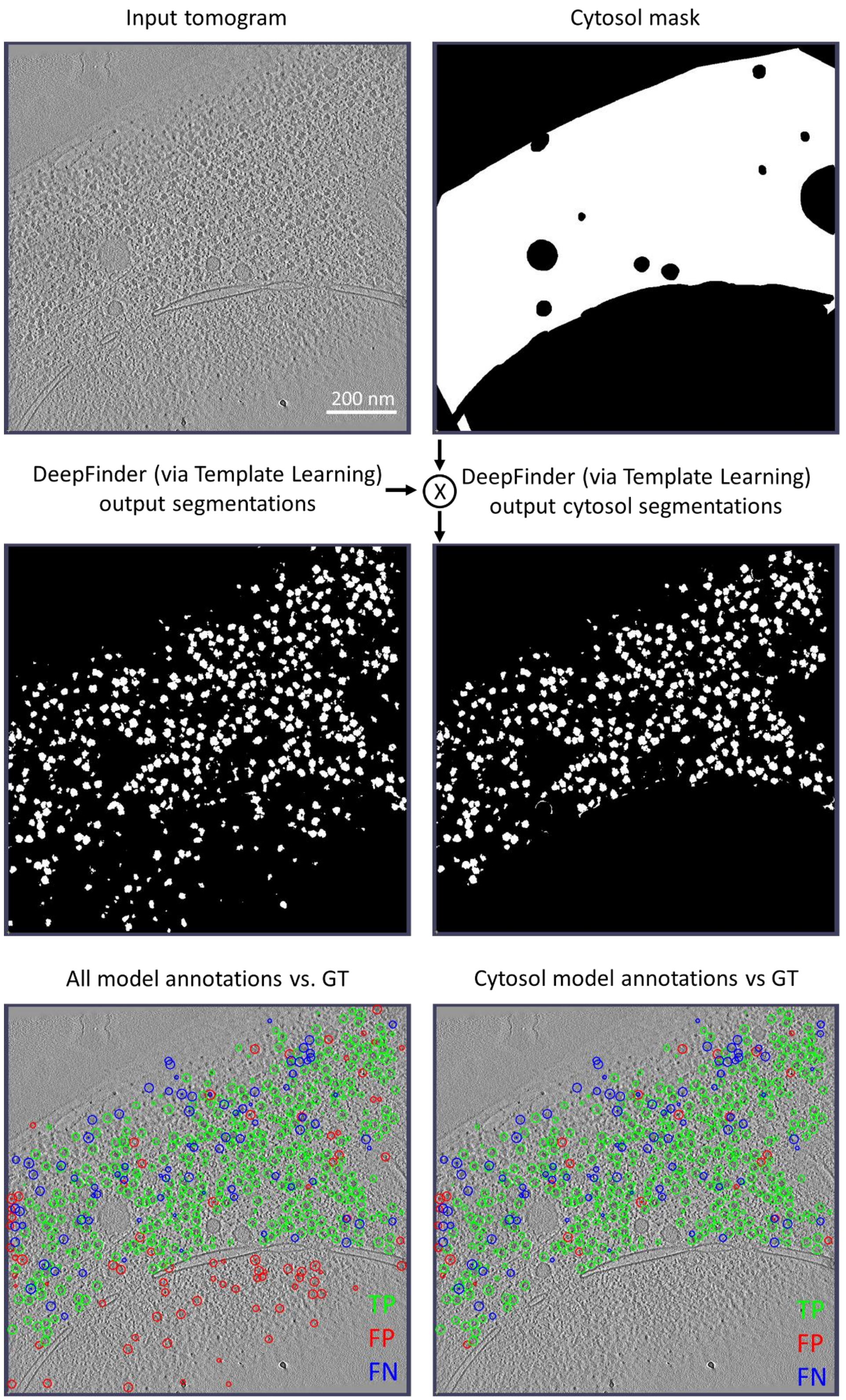

Supplementary Fig. 9: Ribosome segmentation using masks to focus annotations within a defined region of interest. *An exemplar input tomogram and its corresponding cytosol mask*

sourced from EMPIAR-10988 (VPP, Tomo ID TS_0006). Ribosome segmentations were generated through the standard Template Learning pipeline. Coordinates were extracted with and without cytosol masking. The meanshift clustering algorithm was applied with a radius of 10 pixels, followed by a segment threshold of 3000 voxels after clustering. The resulting annotations are color-coded as True Positives (TP), False Positives (FP), and False Negatives (FN), based on comparison with expert-validated annotations (within a 10-pixel radius). At the bottom half of the input tomogram, a nucleus region is shown enclosed below a double membrane. Without using cytosol masking, some ribosome FP annotations may correspond to pre-ribosomal particles, which is expected, since they share subunits with ribosomes. In this example, the total number of ribosomes detected without masking was 1284, which decreased to 1065 with masking. Hence, masking reduced the false positives from 254 to 58 without manual intervention, significantly improving precision without affecting recall, thus enhancing the overall F_1 *score.*

Supplementary Note 2: Descriptive Statistics and Statistical Tests of Template Learning Variations

In this supplementary note, we further analyze the experiments described in Results Section 2 and presented in Fig.2 of the main text (Template Learning Variations). These experiments evaluate different variations of the Template Learning pipeline for annotating ribosomes in situ (EMPIAR-10988, VPP tomograms).

In the main text, we compared these experiments using the median F_1 score to maintain consistency with the assessment done in a previous study [8]. Here, we present and discuss the results in greater detail, first by expanding the plots of precision and recall to include the overall F_1 score (only the median F_1 score was reported in the main text), then including descriptive statistics and statistical tests to assess the statistical significance of these Template Learning variations.

Supplementary Fig. 10 and 11 show the precision and recall plots (same as Fig.2) with the overall F_1 scores added (with and without the cytosol mask). Descriptive statistics for F_1 scores are provided in Supplementary Tables 3 and 4, while statistical test results based on Dunn's test [13] are shown in Supplementary Tables 5 and 6 and illustrated in Supplementary Fig. 12 and 13.

These experiments tested the validity of four key concepts in the Template Learning pipeline:

1- Using multiple templates to capture compositional and flexible variations: In the typical Template Learning pipeline, we incorporated multiple starting templates from the PDB database and augmented them using fast molecular mechanics simulations (coarse-grained Normal Mode Analysis). To assess the validity of this approach, we ran three ablation experiments: using multiple PDB structures (without flexible variations), using a single PDB structure with flexible variations, and using a single PDB structure without flexible variations. The descriptive statistics results confirm that using fewer variable templates reduced the median, mean, and min/max F_1 score ranges. Removing both flexibility and multiple templates significantly reduced performance, demonstrating statistical significance compared to the typical method.

2- Variety of distractors: We hypothesized that using a diverse set of as large as 100 distractors would outperform using fewer or no distractors. The descriptive statistics confirmed a reduction in F_1 scores when fewer (3 out of 100) or no distractors were used, and statistical tests indicated significant differences for both experiments.

3- Simulating molecular crowding: We developed a method, termed the "Tetris algorithm," to simulate molecular crowding, which we hypothesized advantageous over random

molecular placement. Reducing the crowding level to 30% of its original lowered the F_1 score based on the descriptive statistics, a statistically significant reduction.

4- Volumetric vs. atomic templates: We found that using a volumetric template resulted in significantly lower performance compared to multiple and flexibly variable atomic structures.

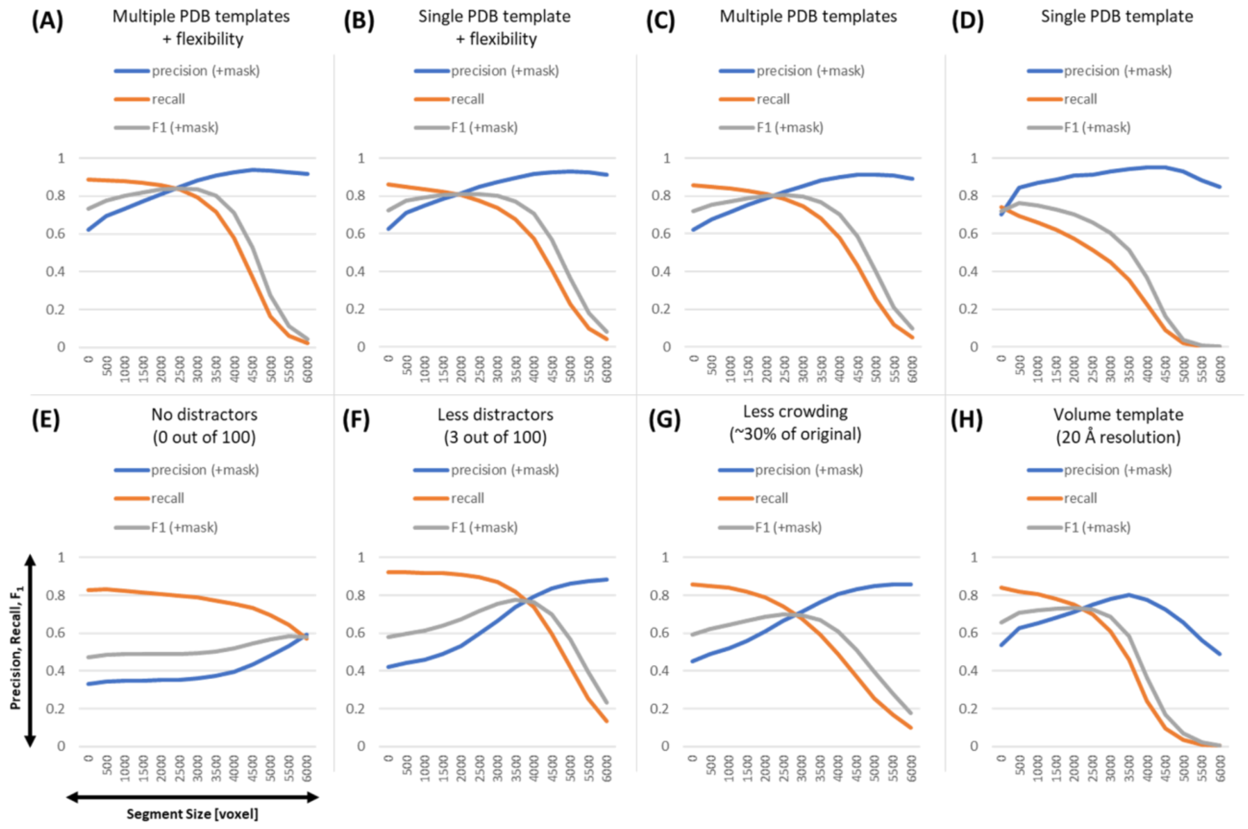

Supplementary Fig. 10: Performance benchmarking of the Template Learning on ribosome annotation on the S. pombe dataset (EMPIAR-10988, 10 VPP tomograms). *A-H Performance measures for 8 variations of Template Learning settings. The curves depict the overall Precision, Recal, and F_1 score against the volume of the segmented region (horizontal axis). Precision and F_1 scores are evaluated with cytosol masking (+mask). Performance in all these experiments was evaluated on $n = 10$ independent tomograms from S. pombe in a single experiment, acknowledging that models trained solely on simulated data yield deterministic results on experimental data, making repeated identical evaluations unnecessary.*

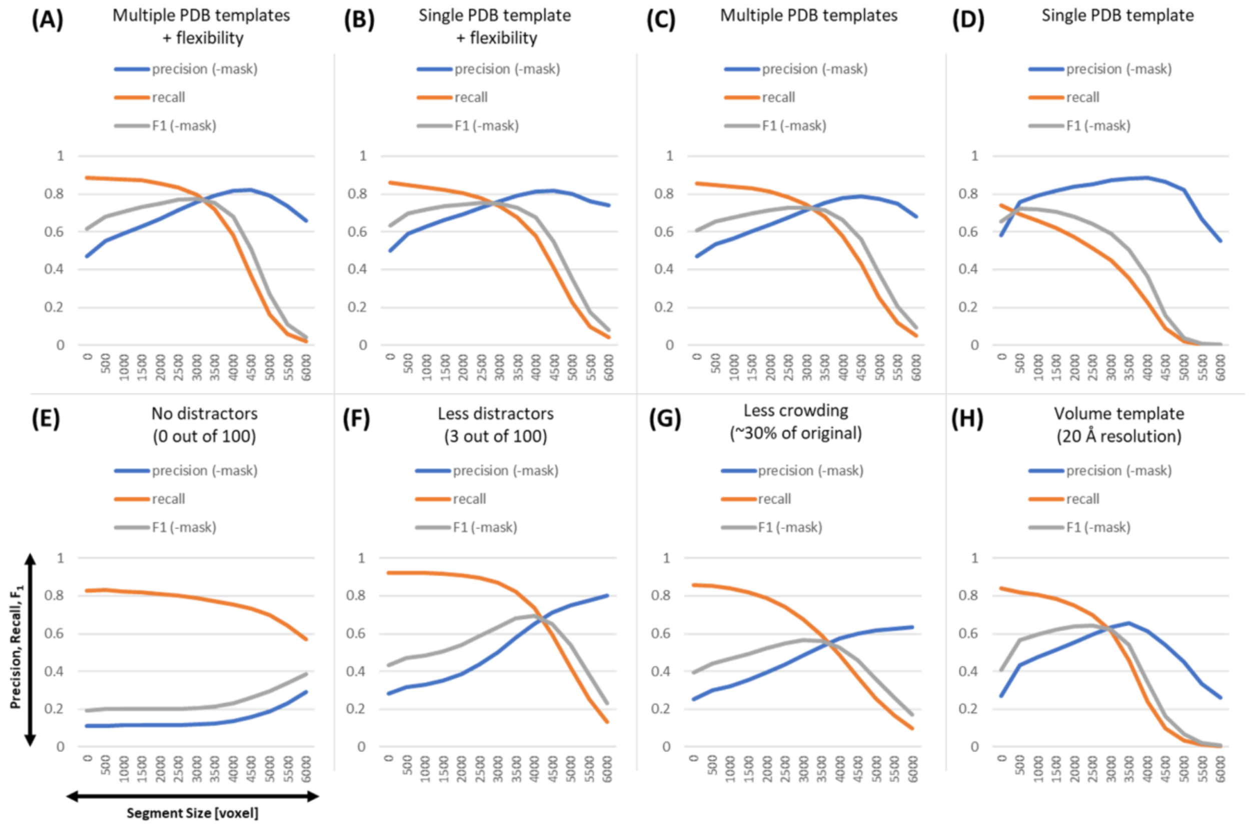

Supplementary Fig. 11: Performance benchmarking of the Template Learning on ribosome annotation on the S. pombe dataset (EMPIAR-10988, 10 VPP tomograms). *A-H Performance measures for 8 variations of Template Learning settings. The curves depict the overall Precision, Recal, and F_1 score against the volume of the segmented region (horizontal axis). Precision and F_1 scores are evaluated without cytosol masking (-mask). Performance in all these experiments was evaluated on n = 10 independent tomograms from S. pombe in a single experiment, acknowledging that models trained solely on simulated data yield deterministic results on experimental data, making repeated identical evaluations unnecessary.*

Supplementary Table 3: Descriptive Statistics (F_1 +mask) of Template Learning Variations

	Multiple PDB templates + flexibility	Multiple PDB templates	Single PDB template	Single PDB template + flexibility	Less crowding (~30% of original)	No distractors (0 out of 100)	Less distractors (3 out of 100)	Volume template (20 A resolution)
Number of values	10	10	10	10	10	10	10	10
Minimum	0.7537	0.6869	0.6139	0.6892	0.5025	0.03547	0.6654	0.5229
Maximum	0.8879	0.8763	0.8091	0.8788	0.7723	0.7129	0.8521	0.8575
Range	0.1342	0.1895	0.1951	0.1896	0.2699	0.6775	0.1867	0.3346
Mean	0.8334	0.7961	0.7331	0.804	0.683	0.5333	0.7623	0.7308
Std. Deviation	0.0465	0.06927	0.06342	0.06037	0.08031	0.2116	0.06106	0.1154

Supplementary Table 4: Descriptive Statistics (F_1 -mask) of Template Learning Variations

	Multiple PDB templates + flexibility	Multiple PDB templates	Single PDB template	Single PDB template + flexibility	Less crowding (~30% of original)	No distractors (0 out of 100)	Less distractors (3 out of 100)	Volume template (20 A resolution)
Number of values	10	10	10	10	10	10	10	10
Minimum	0.6516	0.5927	0.5916	0.6293	0.2711	0.07628	0.4534	0.4793
Maximum	0.8583	0.8347	0.7998	0.839	0.7136	0.4377	0.7939	0.8151
Range	0.2067	0.242	0.2082	0.2097	0.4425	0.3614	0.3406	0.3358
Mean	0.7713	0.7217	0.7044	0.748	0.5137	0.213	0.664	0.6494
Std. Deviation	0.0687	0.09857	0.07315	0.07787	0.1564	0.1191	0.1121	0.1314

Supplementary Table 5: Pairwise Dunn's test (F_1 +mask) of Template Learning Variations

Dunn's test (vs. Typical Template Learning)	Rank sum diff.	Significant?	Summary	P Value
Multiple PDB templates	23	No	ns	0.0604
Single PDB template	55	Yes	****	<0.0001
Single PDB template + flexibility	17	No	ns	0.1651
Less distractors (3 out of 100)	64	Yes	****	<0.0001
No distractors (0 out of 100)	78	Yes	****	<0.0001
Less crowding (30% of original)	42	Yes	***	0.0006
Volume Template (20 A resolution)	51	Yes	****	<0.0001
Mislabeling ~10% of targets as distractors	21	No	ns	0.0864

Supplementary Table 6: Pairwise Dunn's test (F_1 -mask) of Template Learning Variations

Dunn's test (vs. Typical Template Learning)	Rank sum diff.	Significant?	Summary	P Value
Multiple PDB templates	26	Yes	*	0.0338
Single PDB template	39	Yes	**	0.0015
Single PDB template + flexibility	10	No	ns	0.4142
Less distractors (3 out of 100)	66	Yes	****	<0.0001
No distractors (0 out of 100)	78	Yes	****	<0.0001
Less crowding (30% of original)	49	Yes	****	<0.0001
Volume Template (20 A resolution)	49	Yes	****	<0.0001
Mislabeling ~10% of targets as distractors	25	Yes	*	0.0412

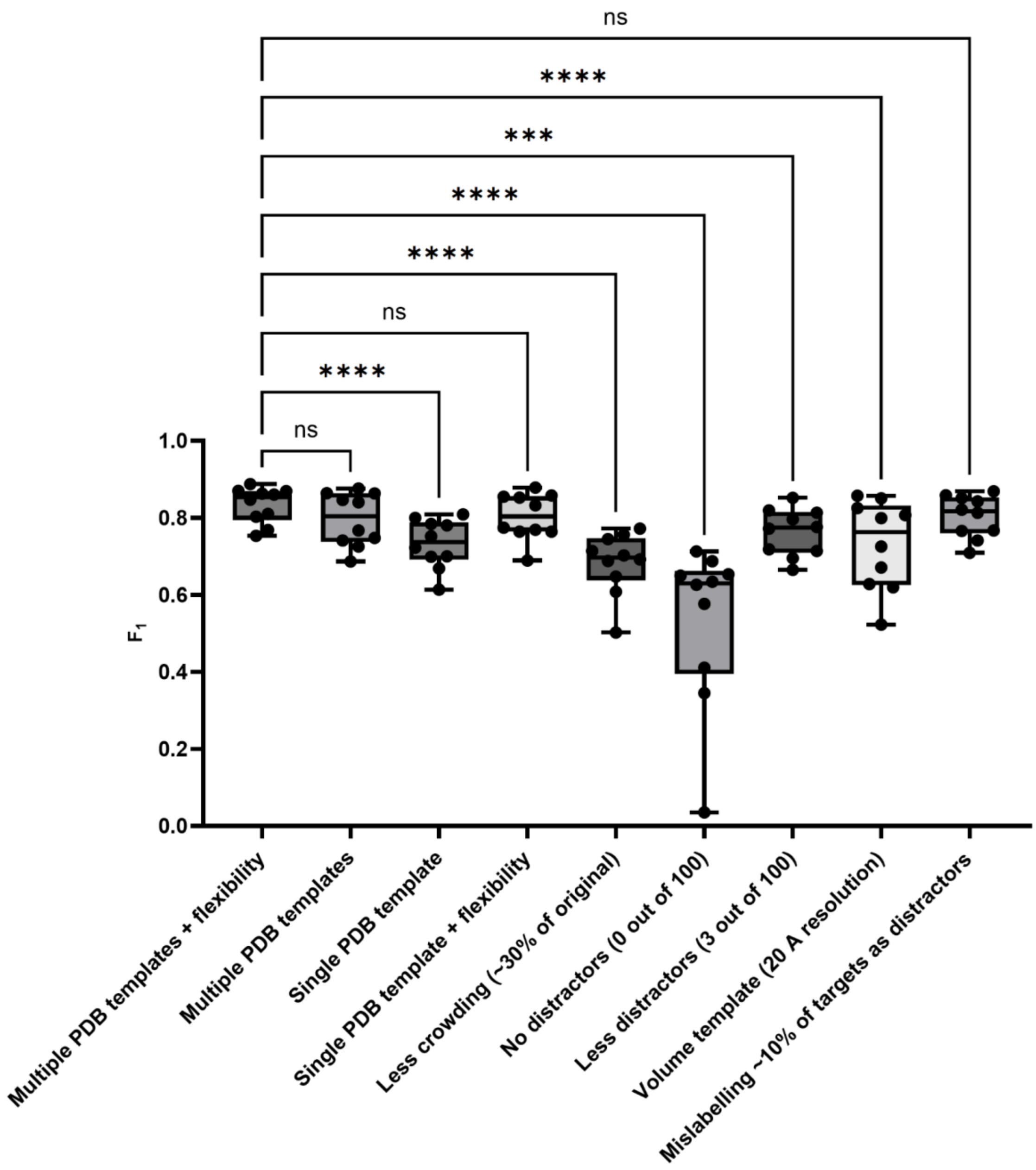

Supplementary Fig. 12: F_1 (+mask) boxplots of Template Learning variations compared using pairwise Dunn's test.

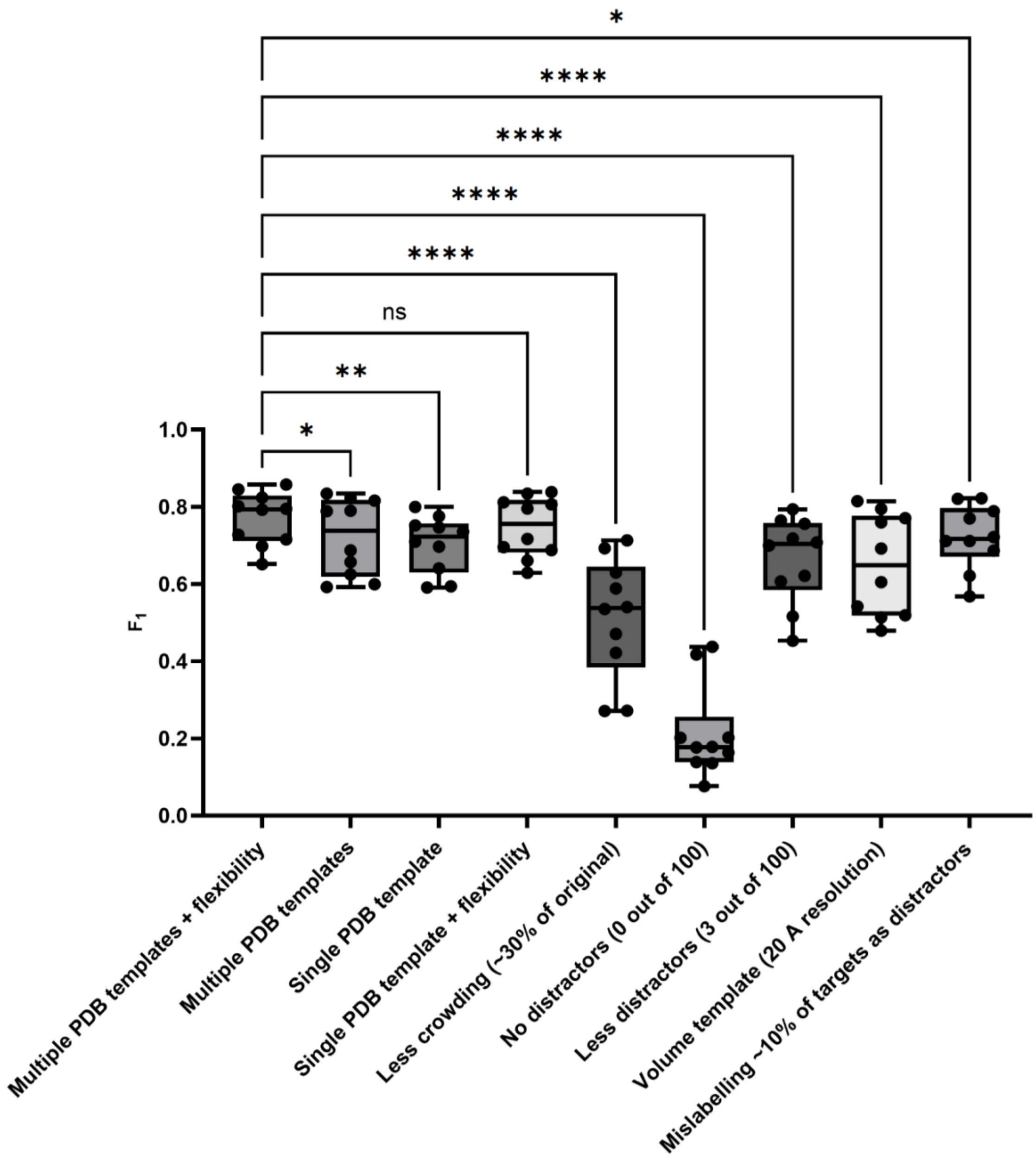

Supplementary Fig. 13: F_1 (-mask) boxplots of Template Learning variations compared using pairwise Dunn's test.

Supplementary Note 3: Impact of Target-Distractor Similarity on Template Learning Performance

This section explores the effect of target-distractor similarity on the performance of Template Learning for particle picking in cryo-electron tomography (cryo-ET) using domain randomization. In the typical Template Learning workflow, we selected 100 structures with various general shapes and sizes to serve as distractors (see Supplementary Fig. 1 for details). These structures were chosen based on prior studies, including the SHREC tomography competition [14] and TomoTwin [15]. In TomoTwin, these distractors were shown to have sufficiently distinct feature representations, allowing Convolutional Neural Networks (CNNs) to distinguish between them. A target structure in Template Learning cannot be similar to all 100 distractors, as the distractors are inherently diverse.

In our data simulation pipeline, each distractor occupies approximately 1% of the total volume in the simulated tomograms. The volumetric density of target molecules and distractors is balanced at a 50-50% ratio. For example, in simulating training data for ribosome and FAS picking, one target molecule (ribosome or FAS) is placed alongside five distractors (see Supplementary Fig. 2). Accordingly, if one of the distractors is similar or identical to the targets, this introduces only about 0.5% "label noise" (1% of 50%). Previous experiments have shown that DeepFinder [16] is robust against this level of label noise.

In the experiment described next, we show the effect of mislabeling some target structures as distractors (i.e., using distractors identical to the targets) during Template Learning on the model's performance of picking these targets in experimental tomograms.

Experiment: Mislabeling Targets as Distractors in Template Learning for Picking Ribosomes *in situ*

We conducted an additional Template Learning variation experiment similar to the experiments used for benchmarking Template Learning for ribosome picking *in situ* (see main text, "Benchmarking Template Learning for Ribosome Picking *In Situ*"). We intentionally mislabeled all the copies corresponding to 18 out of 156 ribosome structures as distractors during training a DeepFinder model for ribosome picking on the EMPIAR-10988 (VPP) dataset. This corresponded to mislabelling ~750 ribosomes out of ~6500 ribosomes used for training as distractors.

The results (Supplementary Fig. 14) show that the DeepFinder model trained on this Template Learning variation maintained a high F_1 score, with only a minor drop of 3% or 7% (measured with and without cytosol masking to remove false positives in noisy regions correspondingly) compared to the typical Template Learning workflow (i.e., before

mislabeling targets as distractors). Statistical tests (see Supplementary Note 2, Supplementary Tables 5 and 6, Supplementary Fig. 12 and 13) show that the drop in performance is either non-statistically significant or with weak statistical significance (with/without cytosol masking). Despite this minor drop in performance, the resulting model remains competitive with state-of-the-art performance in ribosome picking tasks (i.e., compared to the median F_1 scores of 79% for DeePiCt and 83% for DeepFinder trained on experimental annotations).

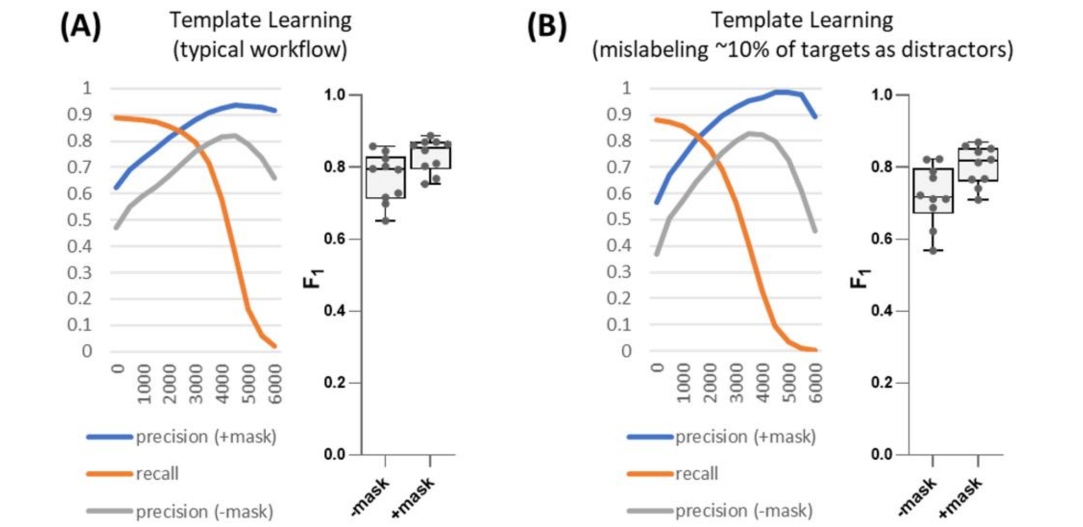

(C)

Deep learning models (DeepFinder) trained solely on simulations – benchmarked on 10/10 experimental tomograms (25k annotations)		
Template Learning experiment	Without mask F_1 median	With cytosol mask F_1 median
Typical workflow	0.79	0.85
Mislabeling ~10% of targets as distractors	0.72	0.82

Supplementary Fig. 14: Assessing the influence of target-distractor similarity on Template Learning performance. ***A:*** *Results of the typical Template Learning workflow applied to ribosome picking on the S. pombe dataset (EMPIAR-10988, VPP tomograms).* ***B:*** *Results from the same dataset (EMPIAR-10988, VPP tomograms), where more than 10% of ribosome structures were intentionally mislabeled as distractors.* ***C:*** *A table comparing the median F_1 scores between the typical workflow (A) and the mislabeling experiment (B). Performance in these experiments was evaluated on $n = 10$ independent tomograms from S. pombe in a single experiment, acknowledging that models trained solely on simulated data yield deterministic results on experimental data, making repeated identical evaluations unnecessary.*

Supplementary Note 4: Subtomogram Averaging of Ribosomes *In Situ*: Template Learning vs. DeePiCt and Expert Annotations

In this supplementary note, we further inspect the ribosome annotations obtained using Template Learning on DEF tomograms of EMPIAR-10988, as discussed in Results - Section 2 of the main text.

The precision, recall, and F_1 score boxplots shown in Fig. 5 of the main text were based on expert-validated ribosome annotations from the publicly available dataset (EMPIAR-10988). Here, we further validate the performance of Template Learning by subtomogram averaging (STA).

1. General Procedure for Ribosome Subtomogram Averaging

We performed the complete STA procedure using Relion V4 using the following settings:

Tilt Series Alignment and CTF Estimation: We imported the tilt series and corresponding alignment matrices from EMPIAR-10988 into Relion V4, and we performed CTF estimations using CTFFind4 [17].

Pseudosubtomogram Reconstruction: We reconstructed ribosomes in volumes of 140^3 pixels (3.3702 Å pixel size, 350 Å particle diameter).

Initial Alignment: We performed a coarse alignment with 10 iterations, using the S. cerevisiae 80S ribosome (EMDB accession EMD-3228, low-pass filtered to 60 Å) as a reference.

3D Refinement: We conducted gold-standard refinement using the resulting initial average as the reference.

***Masking and FSC**:* We did not use masks during subtomogram averaging or for evaluating FSC curves to maintain fair comparisons with previously published structures (EMD 14425 and EMD 14427).

Refinement of Tilt Series Alignment and CTF: After the initial STA refinement, one round of global tilt series alignment and CFT refinement was performed.

2. Particle Averaging of Template Learning Annotations

Based on the procedure explained above, we performed STA for the following annotations:

I. Expert-validated annotations (EMPIAR-10988, the ground truth)
II. All Template Learning annotations
III. True positives (annotations matching expert annotations)

IV. False positives (annotations not agreeing with expert annotations)

(I) STA using the expert-validated annotations (the reproduced ground truth) achieved an initial resolution of 13 Å, which was improved to 11 Å after global tilt series alignment and CTF refinement (see Supplementary Fig. 15). This resolution is in line with the resolution in the original study (EMD-14417, 11 Å). This indicates that our streamlined STA procedure using Relion V4 achieved a similar efficacy to the method used by de Teresa-Trueba et al. in DeePiCt [8]. The authors used Relion V3 with multiple Warp+M rounds for tilt series alignment and CTF estimation.

(II, III) We compared our STA results with the published structure from the same dataset in Supplementary Fig. 15, along with all subtomogram averaging results from Template Learning annotations. These results demonstrate that Template Learning annotations provided subtomograms those averaging achieve higher resolution (TL) than ones obtained with DeePiCt (True Positives, EMD 14425).

For the false positives, we performed a post-alignment classification followed by selecting one class for gold-standard refinement (Supplementary Fig. 16), revealing that 21% of the false positives (~1.5k out of ~7k annotations) converged to a ribosome structure, achieving a resolution of ~18 Å (Supplementary Fig. 15). These results indicate that Template Learning identifies additional true positives missed by the expert annotations.

3. Closer Examination of False Negatives Reveals Lower Resolution Particles Compared to Template Learning True Positives

To explore, why some expert-validated particles were missed by Template Learning resulting in false negatives, we performed STA experiments to compare them with true positives. As demonstrated in the previous section, the true positives identified by Template Learning achieved an STA resolution of around 11 Å. However, since the number of true positive particles (~18.6k) is roughly three times greater than that of false negatives (~6.3k), a direct resolution comparison between the two sets will not be representative. To address this issue, we conducted three independent experiments, each time randomly selecting a subset of ~6.3k particles from the true positives for STA, and compared the results to those from the false negatives. Each subtomogram averaging experiment was performed independently and followed the gold standard procedure outlined earlier.

The results, illustrated in Supplementary Fig. 17, show that the resolution of the Template Learning false negatives (TL_FN) is ~5 Å lower than true positive subsets (TL_TP_Subsets).

Discussion and Conclusion

STA of the Template Learning annotations of ribosomes achieved a resolution comparable to that of expert-validated annotations and, notably, a much higher resolution than previously reported findings based on supervised deep learning (DeePiCt). Interestingly, although 28% of Template Learning's annotations did not match the expert annotations, excluding these so-called false positives did not improve the overall resolution (Supplementary Fig. 15, compare TL and Ground Truth reproduced). This suggests that some of the discrepancies result from true positives missed by expert validators or the inclusion of heterogeneous particles in the expert-validated annotations.

Further analysis of the false positives revealed that 21% (~1.5k particles) converged into a ribosome structure, achieving a resolution of ~18 Å, indicating that Template Learning contributed an additional ~6% to the total ribosome annotations beyond the expert-validated 25.9k particles.

Moreover, our comparison of Template Learning's true positives to false negatives highlights that subsets of true positives (containing the same number of particles as the false negatives) consistently achieved ~5 Å better resolution than the false negatives. This indicates that the fraction of the ground truth annotations that was not picked by Template Learning is indeed more structurally heterogeneous.

These findings demonstrate that while expert-validated annotations are valuable for benchmarking particle-picking methods, they might be prone to selection biases. Our comparisons of STA of expert annotations and Template Learning annotations for nucleosomes *in situ* further support this conclusion (see Supplementary Note 4), reinforcing the robustness of Template Learning in identifying more consistent and higher-resolution particles.

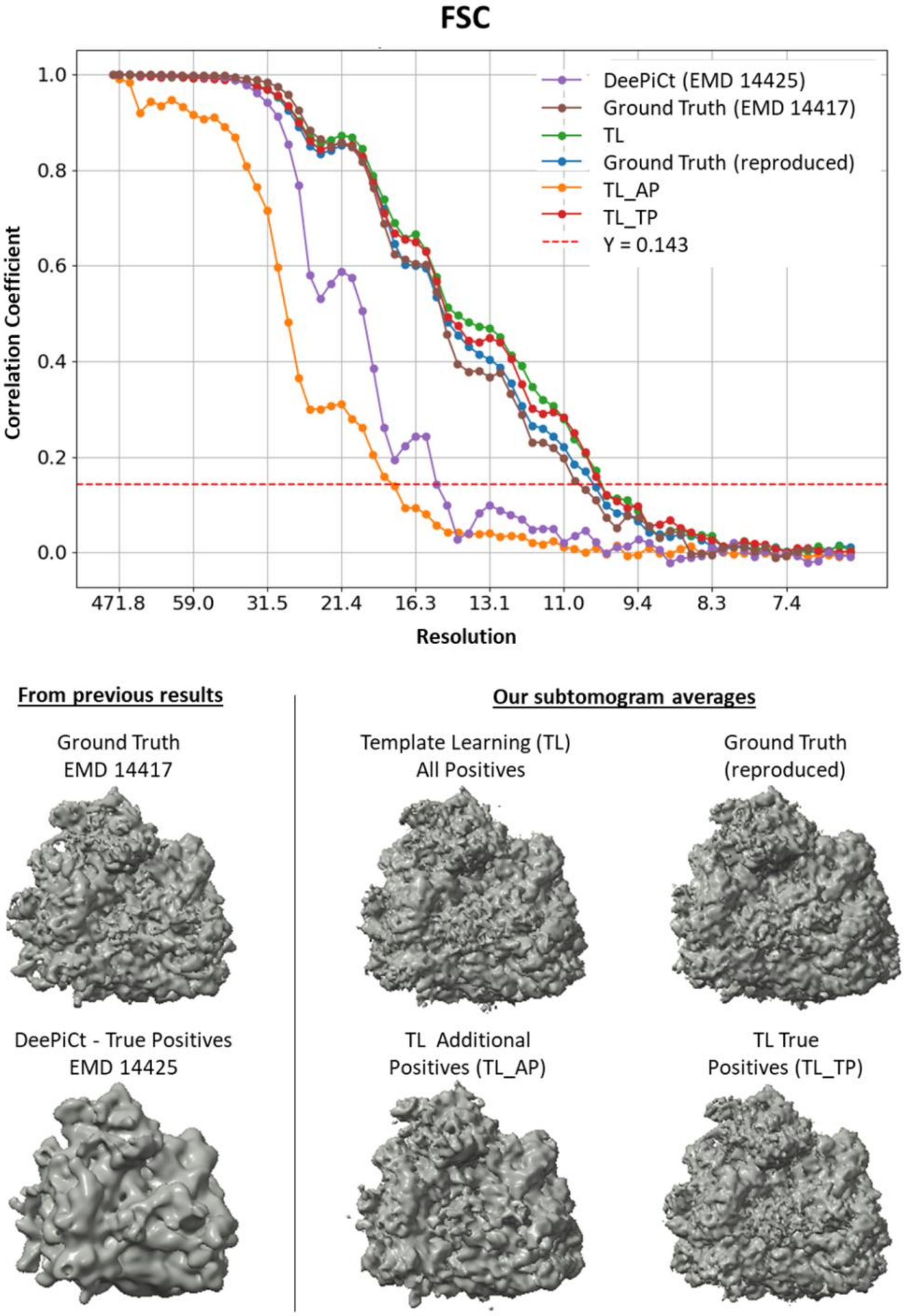

Supplementary Fig. 15: Subtomogram averages and FSC curves for ribosome annotations from the S. pombe dataset (EMPIAR-10988, DEF tomograms). *This figure compares previously published subtomogram averages from expert-validated annotations (Ground Truth) and DeePiCt annotations with our subtomogram averaging results. We present averages from the Ground Truth annotations (reproduced), as well as from Template Learning annotations, including True Positives (those matching the Ground Truth), All Template Learning annotations, and Additional Positives (those not matching the Ground Truth but converging to ribosome structures, procedure shown in Supplementary Fig. 16). The results demonstrate that Template*

Learning achieves higher resolution than DeePiCt and identifies additional true positives missed by the expert annotations.

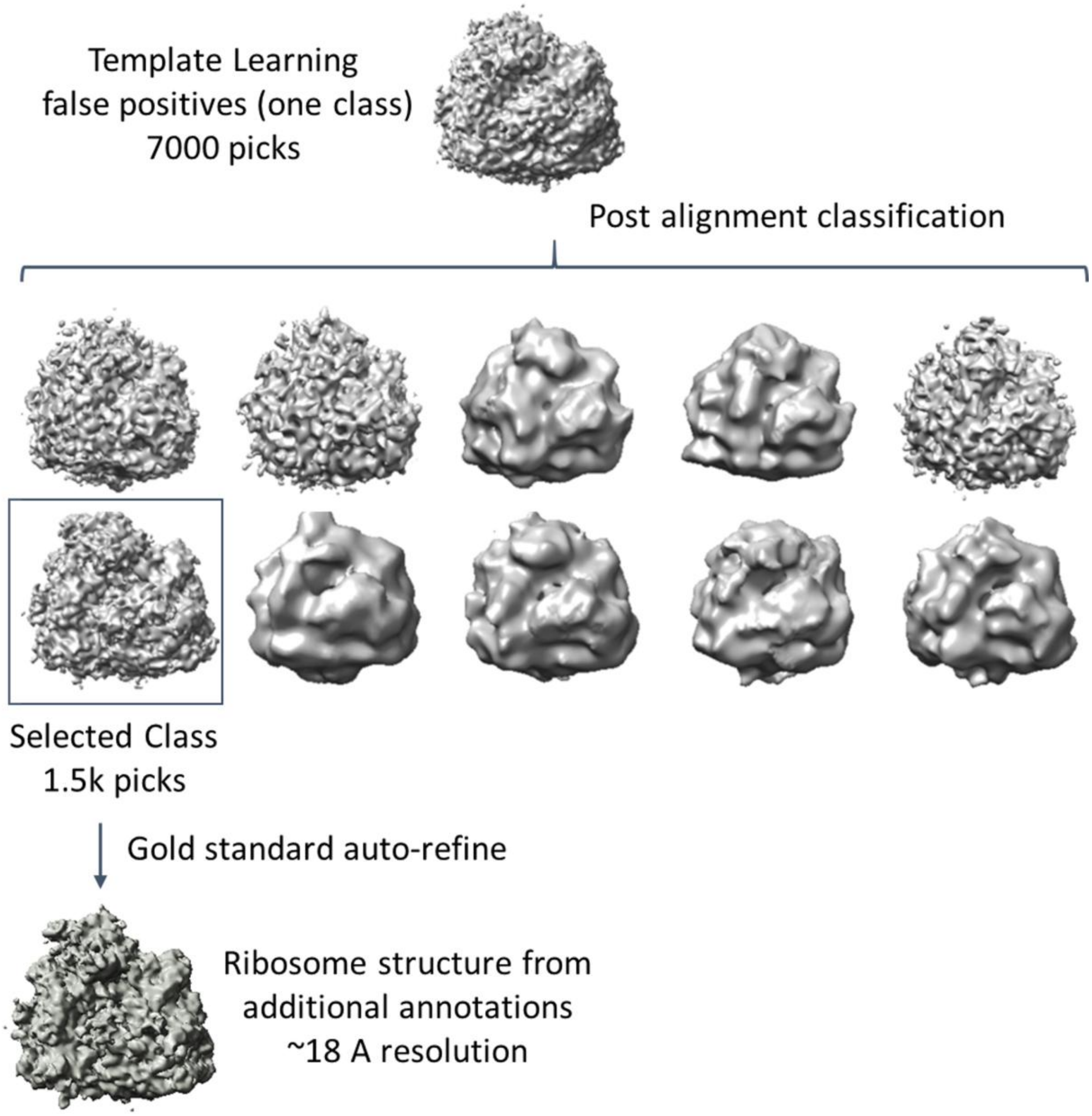

Supplementary Fig. 16: Template Learning can pick additional ribosomes missed by the expert validator. *Subtomogram classification followed by gold-standard refinement of Template Learning annotations for ribosomes on the S. pombe dataset (EMPIAR-10988, DEF tomograms) that initially did not match expert annotations (previously referred to as "false positives"). One of the classes (~21% of particles) converges to a ribosome structure at ~18 Å resolution. The FSC curve of the refined structure is shown in Supplementary Fig. 15.*

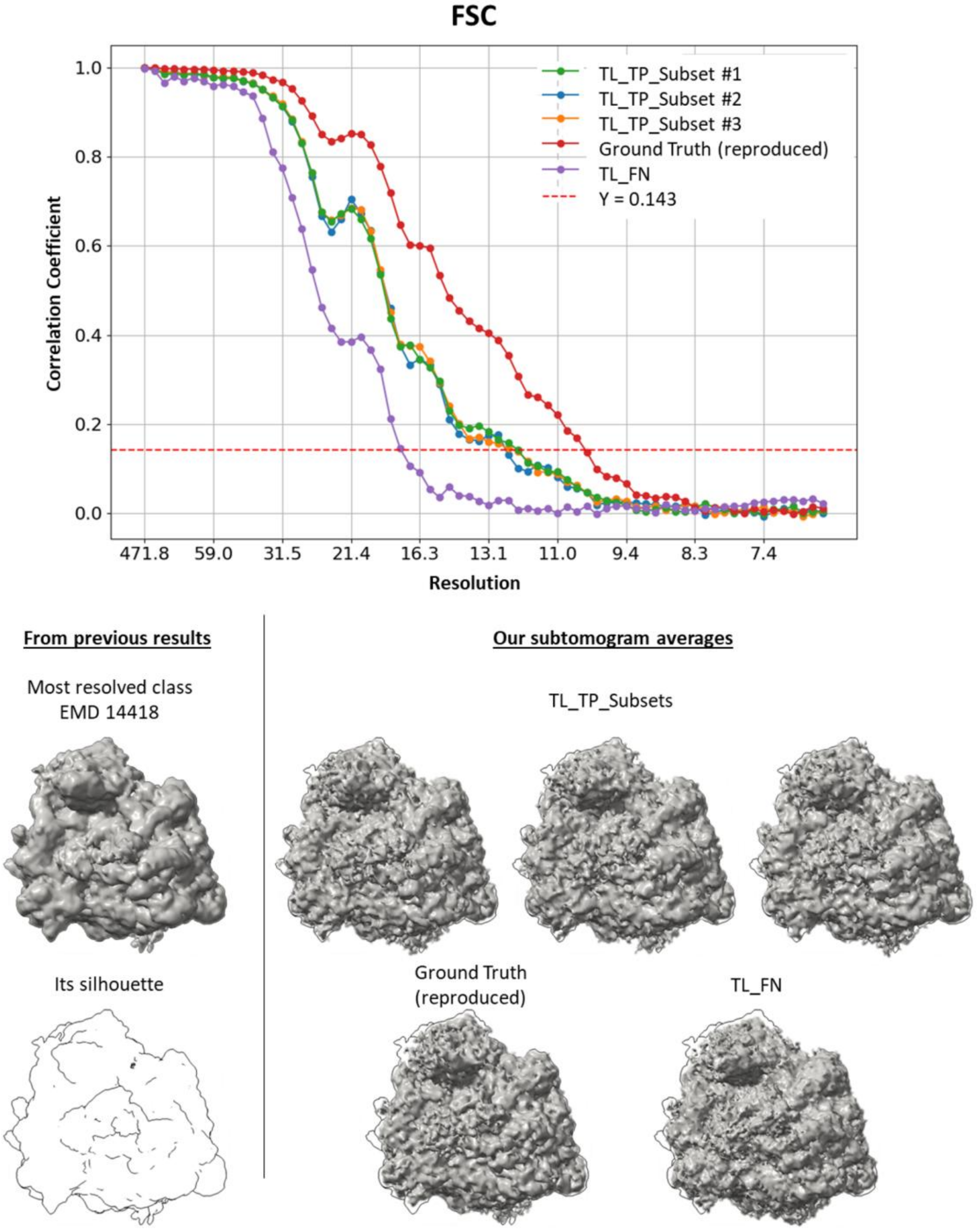

Supplementary Fig. 17: Comparing subtomogram averages and FSC curves Template Learning False Negatives to subsets of True Positives of ribosome annotations from the S. pombe dataset (EMPIAR-10988, DEF tomograms). *This figure compares subtomogram averages and corresponding FSC curves for all expert-validated annotations not identified by Template Learning (i.e., False Negatives, FN, ~6.3k annotations), against averages found from three equal-sized subsets from Template Learning True Positives (~6.3k particles each, TL_TP_Subsets). The subsets were selected through random splits. The results show that the resolution achieved by these subsets is significantly higher than the average from False Negatives*

(improving by ~5 Å). Also, the subset averages show more resolved structural details than the FN annotations. Comparisons are made with the most-resolved class average found from this data (EMD 14418, at 9.3 Å resolution).

Supplementary Note 5: Template Learning Shows Advantages Over Supervised Deep Learning and Template Matching for Annotating Nucleosomes *In Situ*

In this supplementary note, we apply Template Learning to annotate nucleosomes *in situ* using a tomogram representing a cryo-section of Drosophila embryonic brain shown in Supplementary 5.1. This tomogram contains the chromatin domain located in the vicinity of the nuclear envelope as well as a region of nucleolus and cytoplasm. This tomogram, previously analyzed to study nucleosome dynamics in cells [18], was only partially annotated in earlier studies. The raw data pixel size was 2.2 Å without binning, while previous analyses of particle picking and subtomogram averaging were conducted at binning 2 (4.4 Å/pixel). It was used to investigate nucleosome conformational variability [19,20]. In those prior studies, ~650 nucleosomes were manually picked—a challenging task due to the poor performance of traditional 3D template matching methods and the limited number of annotations required for training a deep learning model like DeepFinder. These manually-picked particles were validated by template-free STA that resulted in a characteristic nucleosome shape with approximately 20 Å resolution (EMD-12699; EMPIAR-10679).

There are several challenges when manually annotating nucleosomes in cryo-tomograms. The major challenge is the expert's tendency to annotate more side-view nucleosomes, which are visually distinct and easier to recognize. Nucleosomes in other orientations—such as top, down, or tilted views—are more ambiguous, and it is harder to distinguish them with certainty. This forces the expert into either picking nucleosomes with a high degree of certainty, leading to an overrepresentation of side-view nucleosomes, or including all nucleosome-like densities, risking the inclusion of false positives.

In the following experiments, we compare Template Learning with manual annotation, supervised deep learning (DeepFinder), and 3D template matching using subtomogram averaging.

To ensure a fair comparison between methods, we trained DeepFinder models in two ways: first, using the ~650 manual annotations on the experimental data, and second, using Template Learning simulations, as described in the main text (Results section on nucleosome annotation), but at a similar pixel size to the experimental data. We applied 3D template matching using PyTom with the same parameters as in the main text for *in vitro* nucleosomes but adapted to the new pixel size (4.4 Å) and a larger box size (44 vox^3 compared to 22 vox^3).

The score maps (Supplementary Fig. 19) indicate that the Template Learning-trained model generated fewer false positives outside the chromatin region compared to

supervised DeepFinder and 3D template matching. To extract annotations for subtomogram averaging, for each method, we performed a bulk removal of picks outside the chromatin region using the eraser tool in the Eman2 tomography viewer. This procedure, discussed in more detail in Supplementary Note 1, serves as a simple alternative to generating custom masks. No picks inside the chromatin region were removed, to avoid introducing bias into the comparison of the methods. Following this step, the highest-scoring 2000 annotations from each method were extracted for averaging. To maintain consistency with previously published results based on manual annotations, we followed the same subtomogram averaging procedure detailed in the earlier study [19].

Visualizations of the segmentation maps, picked particles, and their corresponding averages are shown in Supplementary Fig. 19, while the overlap between expert annotations and those generated by other methods is presented in Supplementary Fig. 20. Supplementary Fig. 21–24 provide representative views of the four nucleosome averages (manual annotation, DeepFinder, Template Learning, and 3D template matching), with a docked canonical nucleosome structure (PDB 2PYO).

Supplementary Fig. 20 shows that the angular distribution of subtomogram averages from manual nucleosome annotations exhibits a bias toward side views, for the reasons stated above. The DeepFinder model trained on manual annotations picked more particles but reproduced this bias, with ~83% of the ~650 manual annotations included in the DeepFinder picks (see Supplementary Fig. 21 for overlaps between the different methods and manual annotations). Template Learning, by contrast, produced a more balanced angular distribution, with ~36% of its annotations overlapping with manual annotations. The annotations from Template Learning were more independent and, when averaged, yielded a structure most closely resembling the canonical nucleosome (PDB 2PYO) compared to the other methods (see the structure in Supplementary Fig. 21–24). It is likely that the nucleosomes annotated by the expert but not by Template Learning have larger conformational or compositional variabilities that were not covered by the current simulation method (also see the false negative case of ribosomes in Supplementary Note 4). The 3D template matching method showed the most significant angular distribution bias toward side views, as previously demonstrated in our *in vitro* results (see Fig. 8 in the main text and Supplementary Fig. 8 for details), and had the lowest overlap with manual annotations (~19%). In evaluating the overlap of picks, two picks were considered the same if they fell within a 12-pixel radius (53 Å), a value derived from the 110 Å diameter of the nucleosome.

A video showing mapping back the nucleosome average generated using Template Learning are included in the supplementary of this article.

Conclusion and Discussion:

In conclusion, Template Learning demonstrates advantages over traditional 3D template matching and supervised deep learning methods for picking nucleosomes *in situ*. It reduces human bias in constructing training datasets, offers better coverage of nucleosome orientations, and produces more accurate annotations with fewer false positives, as evidenced by fewer false positives outside the chromatin region compared to supervised DeepFinder and 3D template matching. Additionally, STA of Template Learning picks resulted in densities, more closely resembling the canonical nucleosome.

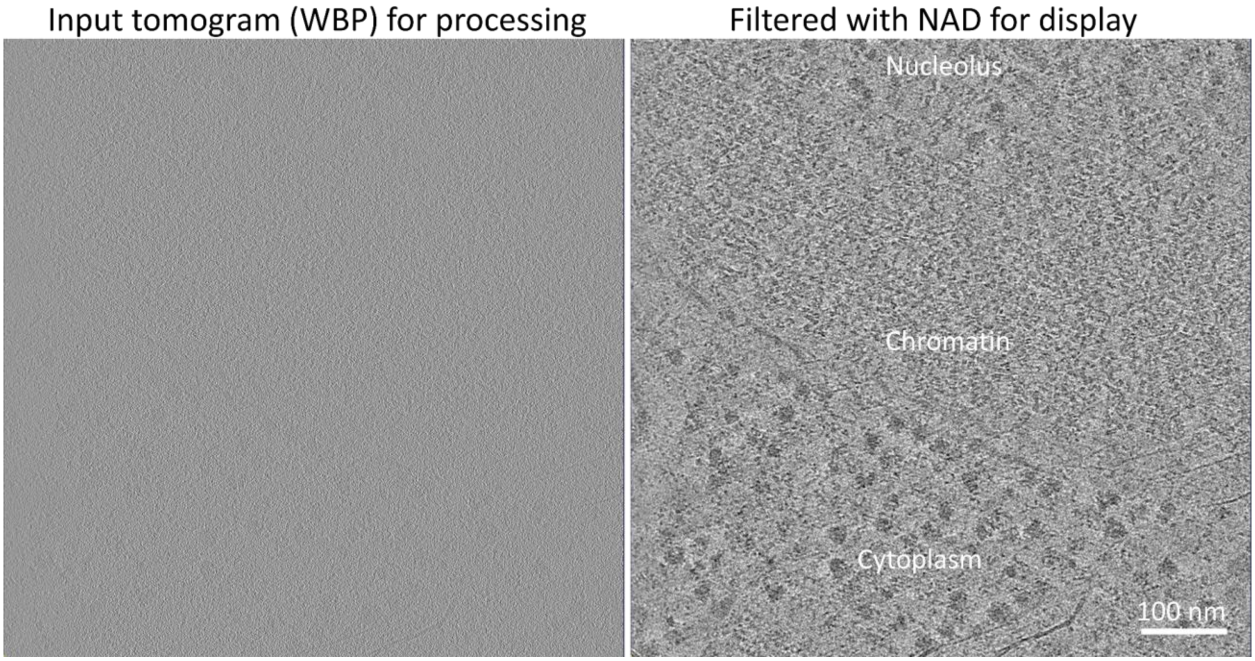

Supplementary Fig. 18: Tomographic central slice of cryo-section of Drosophila embryonic brain used for comparing Template Learning to other techniques in annotating nucleosomes in situ. *Left: a central slice of input tomogram used for picking and subtomogram averaging nucleosomes reconstructed using Weighted Back Projection (WBP) with 3D CTF correction. Right: a central slice of the same tomogram but processed with NAD (Nonlinear Anisotropic Diffusion) filtering, used only for a better display.*

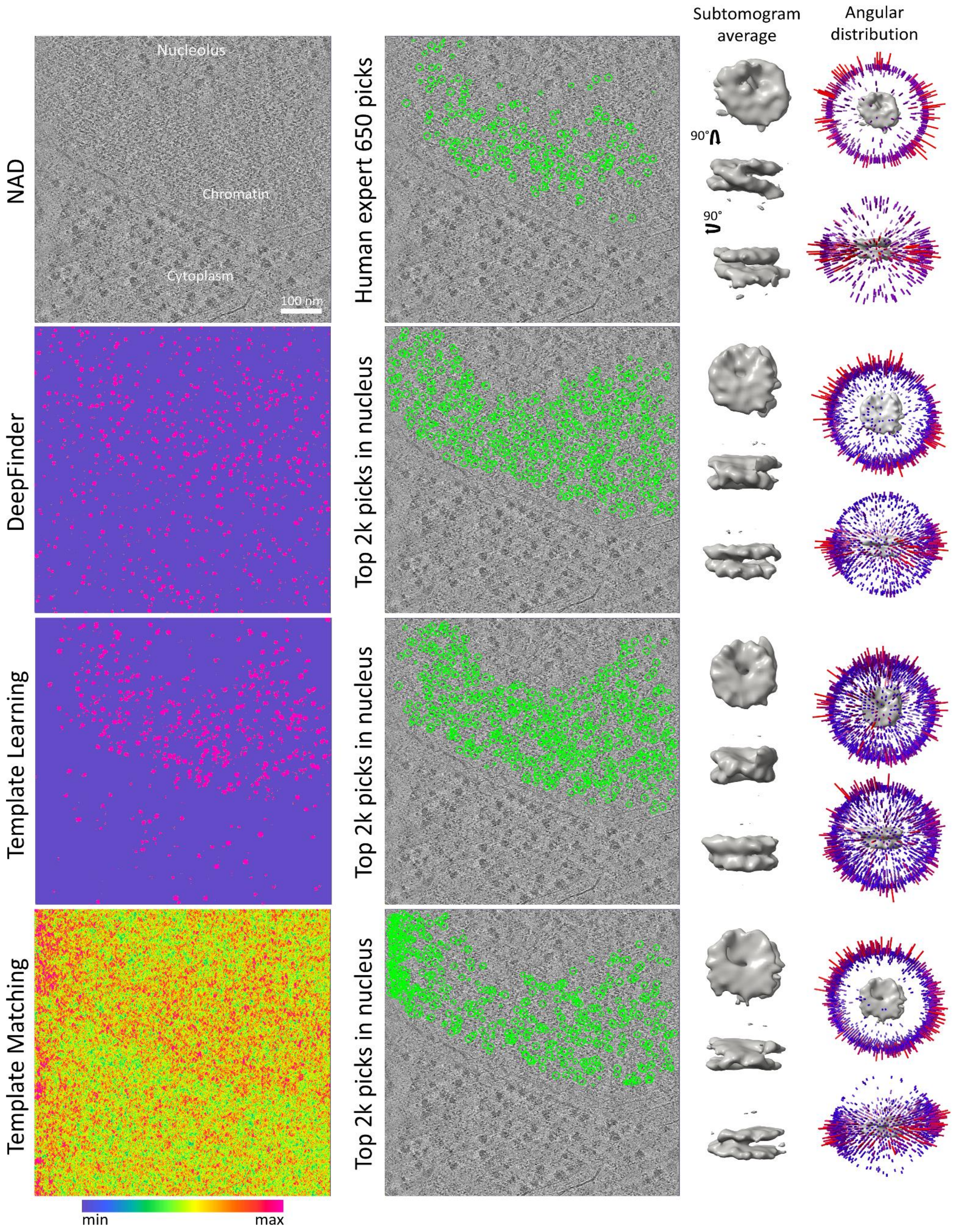

Supplementary Fig. 19: Template Learning outperforms traditional methods in precision and orientational isotropy for annotating nucleosomes in situ. *The right column of the figure*

displays a central slice of the tomogram (reconstructed with WBP and filtered with NAD in IMOD, as shown in Supplementary Fig. 18), alongside the corresponding slices of the score maps (also referred to as segmentation maps) for Template Learning, DeepFinder (trained on the 650 manual annotations), and 3D template matching. The central column shows the same tomogram slice with the picked positions of nucleosomes after manual annotation (Human expert 650 picks), and positions extracted from the score maps after removing false positives from the cytoplasm and nucleolus areas using the Eman "brush eraser" tool, leaving the top score 2k picks from each method. The same settings were used for Eman "bush eraser" for each method. The left column presents the corresponding subtomogram averages and angular distributions. Notice the substantially lower number of false positives in the score map of Template Learning in the cytoplasm and nucleolus regions (right column) and a more isotropic angular distribution (left column) compared to the other methods. The resulting averages are compared. The resulting averages are compared to the canonical nucleosome structure in Supplementary Fig. 21-24.

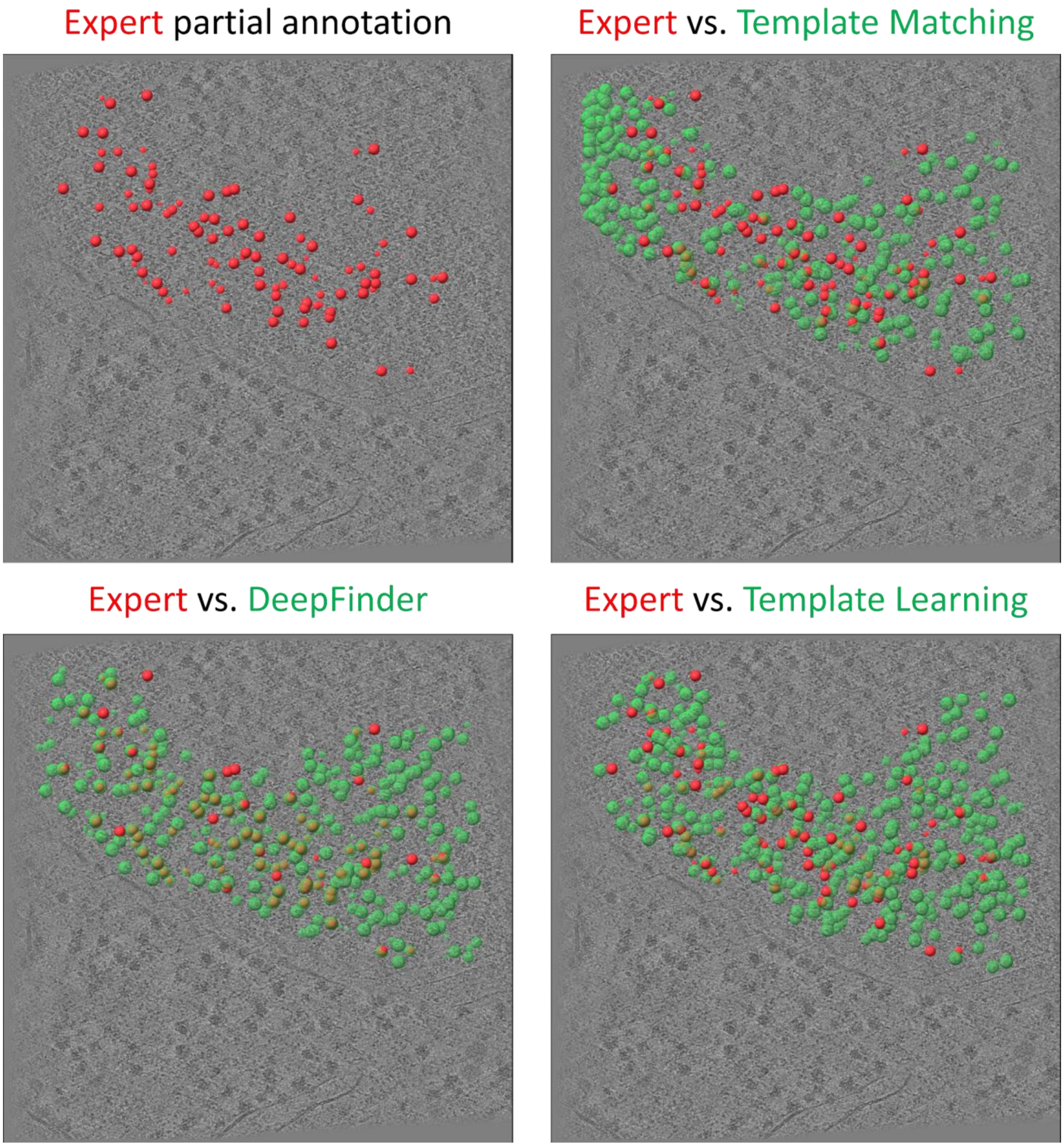

***Supplementary Fig. 20: Overlap between expert partial annotations and those generated by Template Learning, supervised DeepFinder, and 3D template matching on a cryo-tomogram in situ**. Displayed annotations are over a central slice of an in situ cryo-tomogram, displayed in ChimeraX. The overlapping positions (yellow spheres) between the expert and DeepFinder annotations is ~83%, the expert and Template Learning annotations is ~36%, and the expert and Template Matching is ~19%. The annotations of each method were based on the highly scoring 2k annotations in the chromatin region.*

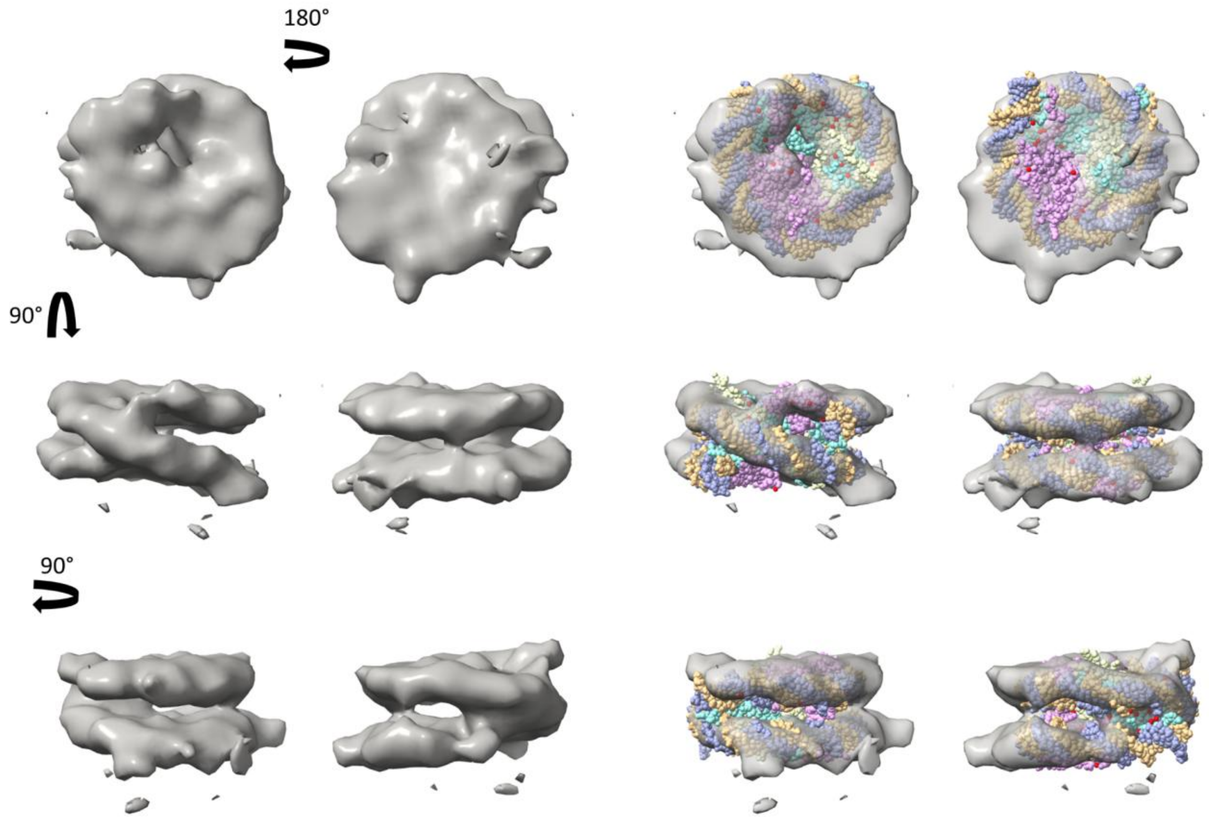

Supplementary Fig. 21: Subtomogram average of nucleosomes from manual annotations on a cryo-tomogram in situ with docked PDB structure (2PYO). *Volume is deposited under the accession code EMD-12699, displayed in ChimeraX with a density threshold of 0.006.*

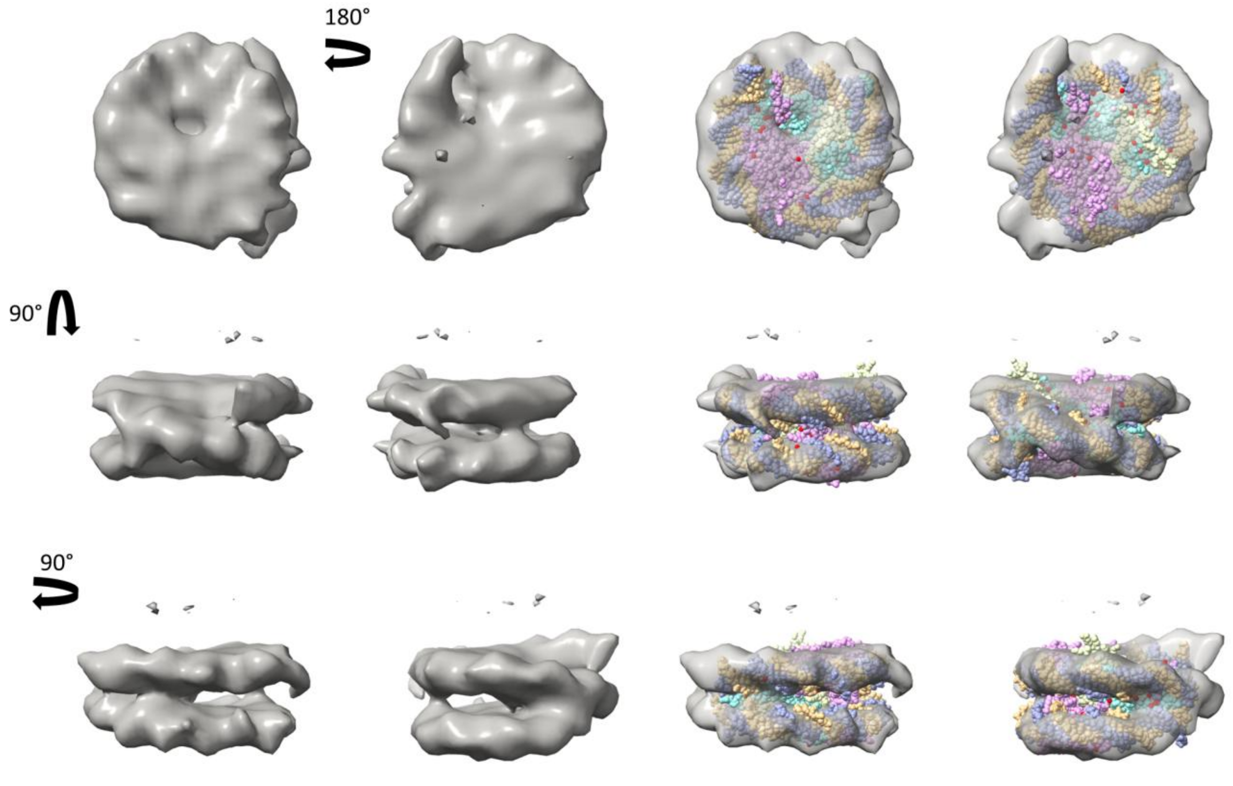

Supplementary Fig. 22: Subtomogram average of nucleosomes from DeepFinder annotations (trained on manual annotations) on a cryo-tomogram in situ with docked PDB structure (2PYO). *Volume is deposited under the accession code EMD-51696, displayed in ChimeraX with a density threshold of 0.006.*

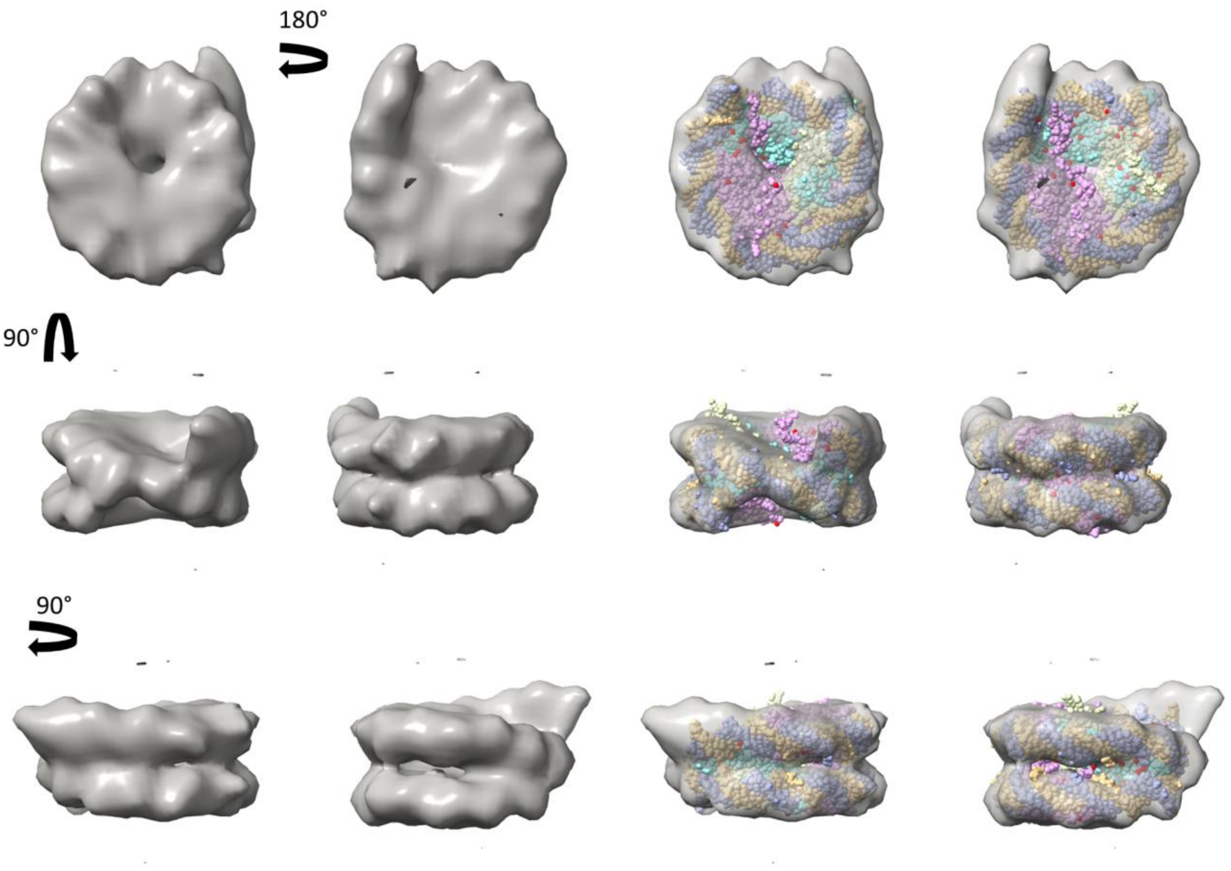

Supplementary Fig. 23: Subtomogram average of nucleosomes from Template Learning annotations on a cryo-tomogram in situ with docked PDB structure (2PYO). *Volume is deposited under the accession code EMD-51694, displayed in ChimeraX with a density threshold of 0.006*.

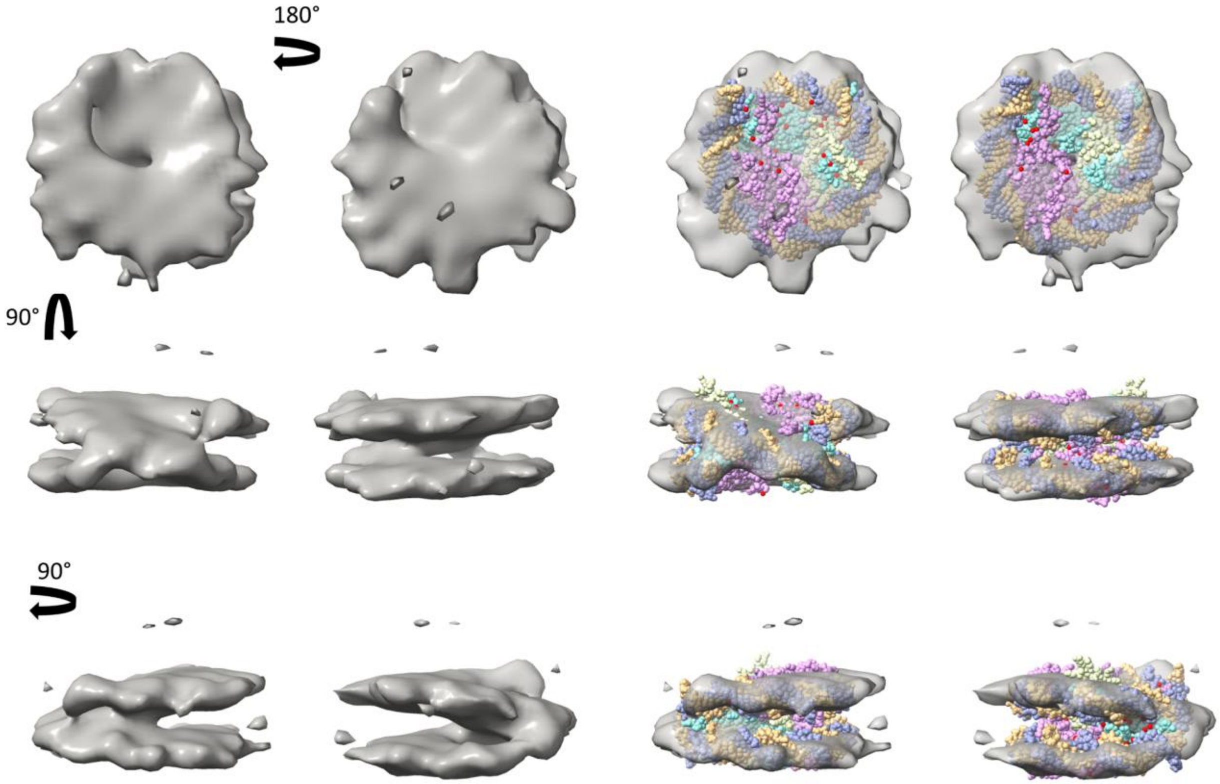

Supplementary Fig. 24: Subtomogram average of nucleosomes from 3D template matching annotations on a cryo-tomogram in situ with docked PDB structure (2PYO). *Volume is deposited under the accession code EMD-51695, displayed in ChimeraX with a density threshold of 0.006.*

References

1. Che, C., Xian, Z., Zeng, X., Gao, X. & Xu, M. Domain randomization for macromolecule structure classification and segmentation in electron cyro-tomograms. in *2019 IEEE International Conference on Bioinformatics and Biomedicine (BIBM)* (IEEE, 2019). doi:10.1109/bibm47256.2019.8983110.
2. Bandyopadhyay, H. *et al.* Cryo-shift: reducing domain shift in cryo-electron subtomograms with unsupervised domain adaptation and randomization. *Bioinformatics* **38**, 977–984 (2022).
3. Purnell, C. *et al.* Rapid Synthesis of Cryo-ET Data for Training Deep Learning Models. *bioRxiv* (2023) doi:10.1101/2023.04.28.538636.
4. Martinez-Sanchez, A., Jasnin, M., Phelippeau, H. & Lamm, L. Simulating the cellular context in synthetic datasets for cryo-electron tomography. *bioRxiv* 2023.05.26.542411 (2023) doi:10.1101/2023.05.26.542411.
5. Jonić, S. & Sorzano, C. Ó. S. Coarse-Graining of Volumes for Modeling of Structure and Dynamics in Electron Microscopy: Algorithm to Automatically Control Accuracy of Approximation. *IEEE J. Sel. Top. Signal Process.* **10**, 161–173 (2016).
6. Chen, M. & Ludtke, S. J. Deep learning-based mixed-dimensional Gaussian mixture model for characterizing variability in cryo-EM. *Nat. Methods* **18**, 930–936 (2021).
7. Tang, G. *et al.* EMAN2: an extensible image processing suite for electron microscopy. *J. Struct. Biol.* **157**, 38–46 (2007).
8. de Teresa-Trueba, I. *et al.* Convolutional networks for supervised mining of molecular patterns within cellular context. *Nat. Methods* **20**, 284–294 (2023).
9. Goddard, T. D. *et al.* UCSF ChimeraX: Meeting modern challenges in visualization and analysis. *Protein Sci.* **27**, 14–25 (2018).
10. Bharat, T. A. M. & Scheres, S. H. W. Resolving macromolecular structures from electron cryo-tomography data using subtomogram averaging in RELION. *Nat. Protoc.* **11**, 2054–

2065 (2016).

11. Kucukelbir, A., Sigworth, F. J. & Tagare, H. D. Quantifying the local resolution of cryo-EM density maps. *Nat. Methods* **11**, 63–65 (2014).
12. Luengo, I. *et al.* SuRVoS: Super-Region Volume Segmentation workbench. *J. Struct. Biol.* **198**, 43–53 (2017).
13. Dunn, O. J. Multiple comparisons using rank sums. *Technometrics* **6**, 241 (1964).
14. Gubins, I. *et al.* SHREC 2020: Classification in cryo-electron tomograms. *Comput. Graph.* **91**, 279–289 (2020).
15. Rice, G. *et al.* TomoTwin: generalized 3D localization of macromolecules in cryo-electron tomograms with structural data mining. *Nat. Methods* **20**, 871–880 (2023).
16. Moebel, E. *et al.* Deep learning improves macromolecule identification in 3D cellular cryo-electron tomograms. *Nat. Methods* **18**, 1386–1394 (2021).
17. Rohou, A. & Grigorieff, N. CTFFIND4: Fast and accurate defocus estimation from electron micrographs. *J. Struct. Biol.* **192**, 216–221 (2015).
18. Eltsov, M. *et al.* Nucleosome conformational variability in solution and in interphase nuclei evidenced by cryo-electron microscopy of vitreous sections. *Nucleic Acids Res.* **46**, 9189–9200 (2018).
19. Harastani, M., Eltsov, M., Leforestier, A. & Jonic, S. HEMNMA-3D: Cryo Electron Tomography Method Based on Normal Mode Analysis to Study Continuous Conformational Variability of Macromolecular Complexes. *Front Mol Biosci* **8**, 663121 (2021).
20. Harastani, M., Eltsov, M., Leforestier, A. & Jonic, S. TomoFlow: Analysis of Continuous Conformational Variability of Macromolecules in Cryogenic Subtomograms based on 3D Dense Optical Flow. *J. Mol. Biol.* **434**, 167381 (2022).
