# Supplementary material for: Template Learning: Deep learning with domain randomization for particle picking in cryo-electron tomography
Source: Nat Commun. 2025 Oct 3;16:8833. doi: 10.1038/s41467-025-63895-0 (PMC12494793; doi:10.1038/s41467-025-63895-0)
Supplement: Supplementary file 2 — Reporting Summary [file 41467_2025_63895_MOESM2_ESM.pdf]

nature portfolio

Corresponding author(s): Mohamad Harastani, Mikhail Eltsov

Last updated by author(s): 27/07/2025

Reporting Summary

Nature Portfolio wishes to improve the reproducibility of the work that we publish. This form provides structure for consistency and transparency in reporting. For further information on Nature Portfolio policies, see our Editorial Policies and the Editorial Policy Checklist.

Statistics

For all statistical analyses, confirm that the following items are present in the figure legend, table legend, main text, or Methods section.

n/a	Confirmed	
☐	☒	The exact sample size (*n*) for each experimental group/condition, given as a discrete number and unit of measurement
☐	☒	A statement on whether measurements were taken from distinct samples or whether the same sample was measured repeatedly
☐	☒	The statistical test(s) used AND whether they are one- or two-sided<br>*Only common tests should be described solely by name; describe more complex techniques in the Methods section.*
☒	☐	A description of all covariates tested
☒	☐	A description of any assumptions or corrections, such as tests of normality and adjustment for multiple comparisons
☐	☒	A full description of the statistical parameters including central tendency (e.g. means) or other basic estimates (e.g. regression coefficient) AND variation (e.g. standard deviation) or associated estimates of uncertainty (e.g. confidence intervals)
☒	☐	For null hypothesis testing, the test statistic (e.g. *F*, *t*, *r*) with confidence intervals, effect sizes, degrees of freedom and *P* value noted<br>*Give P values as exact values whenever suitable.*
☒	☐	For Bayesian analysis, information on the choice of priors and Markov chain Monte Carlo settings
☒	☐	For hierarchical and complex designs, identification of the appropriate level for tests and full reporting of outcomes
☒	☐	Estimates of effect sizes (e.g. Cohen's *d*, Pearson's *r*), indicating how they were calculated

Our web collection on statistics for biologists contains articles on many of the points above.

Software and code

Policy information about availability of computer code

Data collection	The new dataset produced in this work was collected using SerialEM 3.7 or 3.8. (Mastronarde, D.N. 2005. Automated electron microscope tomography using robust prediction of specimen movements. J. Struct. Biol. 152:36-51, doi:10.1016/j.jsb.2005.07.007).<br>Publicly available annotated tomographic reconstructions of S. pombe cryo-FIB lamellae (EMPIAR-10988; de Teresa-Trueba, I., Goetz, S.K., Mattausch, A. et al. Convolutional networks for supervised mining of molecular patterns within cellular context. Nat Methods 20, 284–294 (2023). https://doi.org/10.1038/s41592-022-01746-2) were used for the software benchmarking.
Data analysis	Individual frames were motion-corrected and averaged to form a tilt series using MotionCor2 (V 1.6.3; Zheng, S., Palovcak, E., Armache, JP. et al. MotionCor2: anisotropic correction of beam-induced motion for improved cryo-electron microscopy. Nat Methods 14, 331–332 (2017). https://doi.org/10.1038/nmeth.4193), and CTF was estimated using Gctf (V 1.18; Zhang K. Gctf: Real-time CTF determination and correction. J Struct Biol. 2016 Jan;193(1):1-12. doi: 10.1016/j.jsb.2015.11.003). The tilt series were aligned and reconstructed with IMOD (V 4.11.15; Kremer JR, Mastronarde DN, McIntosh JR. Computer visualization of three-dimensional image data using IMOD. J Struct Biol. 1996 Jan-Feb;116(1):71-6. doi: 10.1006/jsbi.1996.0013.). The aligned frames, reconstructed volume, and annotations were deposited to EMPIAR with the public accession code: EMPIAR-11969.<br>The software for the Template Learning pipelines to training deep learning models on data annotation is available on Github via (https://github.com/MohamadHarastani/TemplateLearning). The software is implemented in Python-3 and depends on a prior user installation of IMOD. It uses CUDA (we used versions 10.2 and 11.4) and Conda (we used version 4.12.0). The code installer automatically uses Conda and PyPi to install packages needed for its functionality with these versions used in this study: Eman2 (V 2.99.47), prody (V 2.4.1), mrcfile (V 1.4.3), scikit-image (V 0.21.0), pyfftw (V 0.13.1), python-parakeet (V 0.4.5) h5py (V 3.8.0). The simulated data was used to training DeepFinder (V 0.2.0; Moebel, E., Martinez-Sanchez, A., Lamm, L. et al. Deep learning improves macromolecule identification in 3D cellular cryo-electron tomograms. Nat Methods 18, 1386–1394 (2021). https://doi.org/10.1038/s41592-021-01275-4; installed through PyPi entry cryoet-deepfinder).

Particle picking using template matching was performed in PyTom (Chaillet, M.L. et al. Extensive Angular Sampling Enables the Sensitive Localization of Macromolecules in Electron Tomograms. Int. J. Mol. Sci. 2023, 24, 13375. https://doi.org/10.3390/ijms241713375; Hrabe T, et al. PyTom: a python-based toolbox for localization of macromolecules in cryo-electron tomograms and subtomogram analysis. J Struct Biol. 2012 May;178(2):177-88. doi: 10.1016/j.jsb.2011.12.003. Epub 2011 Dec 13. PMID: 22193517.available on Github via https://github.com/SBC-Utrecht/PyTom used up the the commit ID 13712a1). ChimeraX (V 1.5) and IMOD were frequently used for data display. Subtomogram averaging was performed in Relion (V 4.0.0). The experiments reported in this manuscript were performed on Dell Precision 5820, Intel(R) Xeon(R) W-2145 CPU @ 3.70GHz, 96 GB DDR4 RAM, 2 X NVIDIA RTX A6000 or NVIDIA Quadro RTX 8000.

For manuscripts utilizing custom algorithms or software that are central to the research but not yet described in published literature, software must be made available to editors and reviewers. We strongly encourage code deposition in a community repository (e.g. GitHub). See the Nature Portfolio guidelines for submitting code & software for further information.

Data

Policy information about availability of data

All manuscripts must include a data availability statement. This statement should provide the following information, where applicable:

- Accession codes, unique identifiers, or web links for publicly available datasets
- A description of any restrictions on data availability
- For clinical datasets or third party data, please ensure that the statement adheres to our policy

The raw frames, alignment information file of the tomogram, aligned tilt series, reconstructed tomogram, original metadata file and the coordinates of nucleosome annotations from the tomogram using Template Learning and template matching are available via EMPIAR accession codes EMPIAR-11969. Subtomogram averaging results of nucleosomes with annotations using Template Learning and template matching are available on EMDB, with accession code, EMD-19823 and EMD-19825 respectively.

Research involving human participants, their data, or biological material

Policy information about studies with human participants or human data. See also policy information about sex, gender (identity/presentation), and sexual orientation and race, ethnicity and racism.

Reporting on sex and gender	N.A.
Reporting on race, ethnicity, or other socially relevant groupings	N.A.
Population characteristics	N.A.
Recruitment	N.A.
Ethics oversight	N.A.

Note that full information on the approval of the study protocol must also be provided in the manuscript.

Field-specific reporting

Please select the one below that is the best fit for your research. If you are not sure, read the appropriate sections before making your selection.

☒ Life sciences ☐ Behavioural & social sciences ☐ Ecological, evolutionary & environmental sciences

For a reference copy of the document with all sections, see nature.com/documents/nr-reporting-summary-flat.pdf

Life sciences study design

All studies must disclose on these points even when the disclosure is negative.

Sample size	From 500 ml of chicken DT40 cell culture with density of approximately 8-10 X 105 cells/ml, 2 ml of mitotic chromsomes could be isolated with a concentration of around 0.5 mg/ml of DNA. From a single chromosome preparation, up to 20 cryo-grids were prepared. On each chromosome, up to four cryo-ET data collection could be made. Our annotation performed on 1 tomogram demonstrated that the volume contained >30,000 nucleosomes, which is sufficient for assessing particle picking quality, angular distribution of the picked particles, and obtaining a high-quality subtomogram average."
Data exclusions	During data collection, only the chromosomes that were placed on the holes of the cryo-EM grid were chosen for data collection. In addition, chromosome regions with adequate 10 nm gold fiducials were preferred for better alignment and reconstruction.
Replication	The training of DeepFinder on simulations resulting from the Template Learning pipeline was repeated 14 times through the manuscript and gives very consistent results. The training, since ground truth information is used, converges for all the tested variations using the default training value of 100 epochs. The replication of the results using different cross-validation splits was necessary when incorporating experimental data for training, i.e., fine tuning DeepFinder models on subset of the annotated tomograms. These experiments were replicated in a three cross-validation setting and reported in the manuscript.

Randomization	Most of the benchmarking of Template Learning was performed on a complete datasets without excluding or selecting tomograms randomly. The experiments that required data splitting were done at random. During subtomogram averaging in RELION V4, particles were randomly divided into two half datasets by the software following gold-standard procedures in subtomogram averaging.
Blinding	Researchers were not blinded during data recording or analysis. However, all the results reported in the manuscript were performed using standardized metrics (i.e., Recall, Precision and F1 scores) and are independent of subjective judgments.

Reporting for specific materials, systems and methods

We require information from authors about some types of materials, experimental systems and methods used in many studies. Here, indicate whether each material, system or method listed is relevant to your study. If you are not sure if a list item applies to your research, read the appropriate section before selecting a response.

Materials & experimental systems

n/a	Involved in the study
☒	☐ Antibodies
☐	☒ Eukaryotic cell lines
☒	☐ Palaeontology and archaeology
☒	☐ Animals and other organisms
☒	☐ Clinical data
☒	☐ Dual use research of concern
☒	☐ Plants

Methods

n/a	Involved in the study
☒	☐ ChIP-seq
☒	☐ Flow cytometry
☒	☐ MRI-based neuroimaging

Eukaryotic cell lines

Policy information about cell lines and Sex and Gender in Research

Cell line source(s)	DT 40 (American Type Culture Collection [ATCC] CRL-2111)
Authentication	None of the authentication procedures was applied
Mycoplasma contamination	Negative
Commonly misidentified lines (See ICLAC register)	*Name any commonly misidentified cell lines used in the study and provide a rationale for their use.*

Plants

Seed stocks	*Report on the source of all seed stocks or other plant material used. If applicable, state the seed stock centre and catalogue number. If plant specimens were collected from the field, describe the collection location, date and sampling procedures.*
Novel plant genotypes	*Describe the methods by which all novel plant genotypes were produced. This includes those generated by transgenic approaches, gene editing, chemical/radiation-based mutagenesis and hybridization. For transgenic lines, describe the transformation method, the number of independent lines analyzed and the generation upon which experiments were performed. For gene-edited lines, describe the editor used, the endogenous sequence targeted for editing, the targeting guide RNA sequence (if applicable) and how the editor was applied.*
Authentication	*Describe any authentication procedures for each seed stock used or novel genotype generated. Describe any experiments used to assess the effect of a mutation and, where applicable, how potential secondary effects (e.g. second site T-DNA insertions, mosiacism, off-target gene editing) were examined.*
